# Association mapping of starch chain length distribution and amylose content in pea (*Pisum sativum* L.) using carbohydrate metabolism candidate genes

**DOI:** 10.1186/s12870-017-1080-9

**Published:** 2017-08-01

**Authors:** Margaret A. Carpenter, Martin Shaw, Rebecca D. Cooper, Tonya J. Frew, Ruth C. Butler, Sarah R. Murray, Leire Moya, Clarice J. Coyne, Gail M. Timmerman-Vaughan

**Affiliations:** 1 The New Zealand Institute for Plant & Food Research Limited, PO Box 4704, Christchurch, New Zealand; 2 USDA-ARS Western Regional Plant Introduction Station, 59 Johnson Hall, WSU Pullman, Pullman, Washington WA 99164-6402 USA

**Keywords:** *Pisum sativum*, Amylopectin, Amylose, Chain length distribution, Association mapping, Candidate genes

## Abstract

**Background:**

Although starch consists of large macromolecules composed of glucose units linked by α-1,4-glycosidic linkages with α-1,6-glycosidic branchpoints, variation in starch structural and functional properties is found both within and between species. Interest in starch genetics is based on the importance of starch in food and industrial processes, with the potential of genetics to provide novel starches. The starch metabolic pathway is complex but has been characterized in diverse plant species, including pea.

**Results:**

To understand how allelic variation in the pea starch metabolic pathway affects starch structure and percent amylose, partial sequences of 25 candidate genes were characterized for polymorphisms using a panel of 92 diverse pea lines. Variation in the percent amylose composition of extracted seed starch and (amylopectin) chain length distribution, one measure of starch structure, were characterized for these lines. Association mapping was undertaken to identify polymorphisms associated with the variation in starch chain length distribution and percent amylose, using a mixed linear model that incorporated population structure and kinship. Associations were found for polymorphisms in seven candidate genes plus Mendel’s *r* locus (which conditions the round versus wrinkled seed phenotype). The genes with associated polymorphisms are involved in the substrate supply, chain elongation and branching stages of the pea carbohydrate and starch metabolic pathways.

**Conclusions:**

The association of polymorphisms in carbohydrate and starch metabolic genes with variation in amylopectin chain length distribution and percent amylose may help to guide manipulation of pea seed starch structural and functional properties through plant breeding.

**Electronic supplementary material:**

The online version of this article (doi:10.1186/s12870-017-1080-9) contains supplementary material, which is available to authorized users.

## Background

The pulses, or grain legumes, are a subset of the legumes (Fabaceae) that accumulate starch as a storage component of the seeds. The pulses include economically important species such as pea (*Pisum sativum* L.), chickpea (*Cicer arietinum* L.), common bean (*Phaseolus vulgaris*) and lentil (*Lens culinaris* Medik.). The composition and nutritional qualities of pulses have been the subject of recent reviews [1, 2]. Pulses are relatively high in protein (16–35% dry weight) and carbohydrates (49–68%) and relatively low in oil (0.5–7%), minerals and vitamins. The carbohydrate composition of pulses includes starch (22–45%), dietary fiber (15–32%) and oligosaccharides (α-galactosides). Pulses also contain phytic acid and phenolic compounds which contribute to their nutritional qualities. Nutritionally, the pulses are characterized by the slow digestibility of their carbohydrate, which gives them relatively low glycaemic index (GI) values for carbohydrate-containing foods [2].

Starch is a polymer of α-1,4-glucose moieties with occasional α-1,6-glycosidic branches. Amylose (which is primarily a linear molecule of α-1,4-glycosidic linkages with approximately 0.1% α-1,6-glycosidic branches, molecular weight 5 × 10^5^ to 10^6^) and amylopectin (which is a branched molecule with approximately 5–6% α-1,6-glycosidic branches, molecular weight 10^7^ to 10^8^) are the two classes of starch [3, 4]. The branching patterns of amylopectin have been described in terms of A, B and C chains. C chains contain the only reducing end in intact amylopectin and provide the central chain from which B chains branch via α-1,6-glycosidic branches. The A chains are the outer chains, and these branch off the B chains also via α-1,6-glycosidic branches. Within the starch granule, starch molecules pack together to form a semi-crystalline structure that is based on the organization of double helices from amylopectin short chains into crystalline lamellae. Two forms have been described based on x-ray diffraction, the A-form that is found in wild-type maize seeds and the B-form that is found in wild-type potato tubers. The crystalline structure of pea starch has been described as a C-form starch, consisting of both A- and B-types [5].

A major aim of the body of research on starch biochemistry and genetics has been developing the ability to manipulate starch structure [6], thereby influencing functional properties and the subsequent uses for starch in food and industrial applications [7, 8]. Making changes to amylopectin chain length distribution (CLD) is one approach. Research on starch from different botanical sources has indicated that differences in amylopectin CLD influence functional properties such as gelatinization, enthalpy change and pasting [9]. Within individual species, effects of modifying CLD on functional properties have also been shown. In both rice and maize, variation in the *ae* gene (amylose extender, starch branching enzyme IIb, SBEIIb) resulted in altered CLD and gelatinization properties [10, 11]. In another rice example using recombinant inbred lines, Luo et al. [12] compared the effects of *indica* versus *japonica* alleles at six genes on CLD and starch functional properties. They showed that the *SSIIa japonica* allele, compared to the *SSIIa indica* allele, increased short length chain proportions while decreasing intermediate length chain proportions altering viscosity properties (reduced peak viscosity and breakdown) and reducing gelatinization temperatures. The *SBEIIb japonica* allele also increased short length chain abundance, compared to *SBEIIb indica* and had an effect on pasting and thermal properties, although the effects were less marked than for the *SSIIa* alleles. In pea, different field pea cultivars have been shown to produce seed starch with different structural attributes, leading to variation in functional properties such as swelling power, gelatinization and pasting properties [13, 14].

The effects of six genes in the seed starch biosynthetic pathway on the composition, structure and physico-chemical properties of pea starch have been examined based on studies of a range of mutant lines [15, 16]. Genes at the *rb* (ADP-glucose pyrophosphorylase L1 subunit, AGPL1), *rug3* (plastidial phosphoglucomutase, PGMP) and *rug4* (sucrose synthase, SuSy) loci affect substrate availability, while *rug5* (starch synthase II, StSynII), *lam* (granule bound starch synthase I, GBSSI) and *r* (starch branching enzyme I, SBEI) affect starch polymer biosynthesis and starch branching directly. Bogracheva et al. [5] found from x-ray crystallography that the A-type became more prevalent in the *rug3* mutant, and the B-type became more prevalent in the *r*, *lam* and *rug5* mutants. No A-type starch was detected for *r* mutants. The *lam*, *r* and *rug5* mutations also affected the proportion of the starch that was in amylose versus amylopectin. In comparison with wild type (30% amylose), the *lam* mutation reduced the amylose content to 4–10%, while the *r* and *rug5* mutations increased amylose content to 60–75% and 43–52%, respectively. These mutants also affected the gelatinization temperatures obtained for isolated starch with *rb*, *rug3* and *rug4,* resulting in higher peak temperatures than those of wild type. In terms of naturally occurring allelic variation, *r* locus has the greatest effect on pea phenotypes. Broadly speaking, peas are categorized as field peas (with “round” seeds) or process peas (with “wrinkled” seeds), based on whether their genotype is *R_* or *rr*, respectively.

In a number of species, particularly in the cereals, *Arabidopsis thaliana* and potato, roles have been established for a number of genes that encode proteins involved in starch biosynthesis and metabolism, and hence contribute to starch molecular structure and physicochemical properties [6, 17, 18]. These include enzymes involved in the pathway from sucrose to the precursor molecule ADP-glucose; in the polymerization of α-1,4-glucosyl chains (starch synthase, StSyn); in the addition of α-1,6-glucosyl linkages to produce branched starch (starch branching enzyme, SBE) leading to amylopectin; and in further debranching to rearrange the amylopectin structure (isoamylase, ISA and pullulanase, PUL). Studies of mutant lines in rice and Arabidopsis [19], as well as in vitro studies [20–22], have shown the importance of starch synthases, branching enzymes and isoamylases in determining the branch point and chain length distributions of starch. In their recent review, Sonnewald and Kossman [18] identified 46 *Arabidopsis* genes related to starch metabolism.

Association mapping studies in other plants have also demonstrated association between the properties of starch from sink tissues and allelic variation in starch pathway genes, including genes from the substrate supply, chain elongation, branching and debranching portions of the pathway. In *indica* and *japonica* rice (*Oryza sativa*), association analysis using candidate genes showed that variation in three physicochemical properties (amylose content, gel consistency, and gelatinization temperature) related to eating and cooking quality was associated with allelic variation in starch pathway genes, primarily with the *Waxy* (*Wx*, *GBSSII*) and starch synthase II-3 (*SSII-3)* genes [23]. Genes with minor effects were also identified, involved in substrate availability (two AGPase large subunit isoform genes), polymerization (*SSI*, *SSII*, *SSIII* and *SSIV-2*), branching (*SBE3*) and rearrangement and cleavage of starch branches (*PUL* and *ISA*). These authors confirmed the roles of *SBE3* and *SSII-3* through transgenic studies, showing that either repression (SBE3) or increased expression (*SSII-3*) affected all three properties. In a maize (*Zea mays*) study [24], the effect of allelic variation in six candidate genes known to control starch content identified three that affected amylose content and/or pasting properties. Two of these genes were involved in substrate supply (*sh1*, the major sucrose synthase gene; and *sh2*, the AGPase large subunit gene), with the third involved in amylopectin production (*ae1*, *SBEIIb*). In a more recent study that took a nested association mapping approach [25], associations were detected involving genes encoding sucrose synthase, β-amylase and α-amylase, but associations were not identified for candidates expected to play a major role in determining kernel composition (i.e. *Wx*, *su1* (an isoamylase-type debranching enzyme) and *sh2*). In sorghum, polymorphisms in three candidate genes involved in starch synthesis and amylopectin production (*SSIIa*, *GBSS* and *SBE*) were found to be associated with starch physicochemical properties, including gelatinization temperature and viscosity parameters [26].

Fluorophore-assisted carbohydrate electrophoresis (FACE) is an analytical method for characterising the CLD of starch (summarized in [27]), and the method for starch molecular characterization that has been used in this study. Briefly, to determine CLD using FACE, isolated starch is debranched using either ISA or PUL enzymes, the reducing ends of the debranched starch oligosaccharides are labelled with a charged fluorophore such as 8-aminonaphthalene-1,3,6-pyrenetrisulfonic acid (APTS), the labelled oligosaccharides are separated on a high resolution platform such as capillary electrophoresis with fluorescence detection, and the resulting chromatograms are analysed to provide peak areas as quantitative measures of the relative abundance of oligosaccharides with different numbers of monosaccharide units (degrees of polymerization, DP). Other methods used to characterize starch molecular structure offer greater instrumental and technical difficulties because of the requirement for the starch to be fully dissolved, which requires use of chaotropic agents such as DMSO and LiCl. These methods include nuclear magnetic resonance spectroscopy (NMR) to estimate the ratio of α-1,4 to α-1,6-glycosidic linkages and size exclusion chromatography to estimate molecular weight, radius and size based on various detection methods [8].

Quantitative trait locus (QTL) and association mapping are two approaches for understanding the genetic basis of trait variation, which may result in the identification of molecular markers or sequence polymorphisms that are linked to a causal mutation influencing phenotype. QTL mapping is limited to family-based populations developed by crossing a limited number of parental lines, while association mapping is based on germplasm panels of diverse individual lines. Association mapping panels offer the potential for higher mapping resolution than is obtained from family-based studies resulting in reduced linkage disequilibrium (LD) between trait and marker loci [28]. Association mapping also has the potential to capture a wider range of alleles because of the greater genetic diversity in the germplasm panel. False positives can arise from association mapping in situations where there is undetected population structure or relatedness among the lines in an association mapping panel, resulting in marker-trait association that is the product of population evolutionary processes rather than linkage. Statistical approaches have been developed to identify population structure and relatedness [29, 30] based on genotypes of the contributing lines obtained using a panel of random background molecular markers, permitting the confounding effects of population structure or relatedness on association mapping to be minimized or removed through inclusion in appropriate models. Approaches that have been devised to identify the relatedness of lines in an association mapping panel [28] include structured association using the Bayesian model-based STRUCTURE program producing a Q matrix [30], Principal Components Analysis (PCA) producing a P matrix [29] and estimation of relatedness using a kinship (K) matrix [31].

In this study, a candidate gene association mapping approach has been taken to explore the genetic determination of the variation in CLD in pea. The candidate gene approach has limitations, especially the obvious one that associations will only be identified if the allelic variation contributes to trait variation that is greater than the statistical power of the experiment for detecting an effect. Associations that are detected between a trait and candidate gene polymorphism may reveal the causal variant(s) or the variant(s) that are in LD with the trait genetic determinant(s). There is substantial information available about pea starch synthesis pathways, reinforced by knowledge of starch synthesis in other plant species, facilitating the choice of candidate genes.

This study explores the range of variation that exists in a diverse collection of pea germplasm with respect to starch candidate gene allelic variation, amylose composition of extracted starch, and debranched starch CLD, one measure of the structural properties of pea starch. We describe the allelic variation in 25 pea candidate gene sequences that have been shown to be involved in carbohydrate and starch metabolism, or that are orthologues of sequences that have been shown in other species to be involved in carbohydrate and starch metabolism. Using an association mapping approach, allelic variants have been identified that show a significant association with variation in CLD or percent amylose (%amylose).

## Methods

### Plant material

Accessions for association mapping were chosen from among the USDA-ARS Refined Pisum Core (https://npgsweb.ars-grin.gov/gringlobal/method.aspx?id=492806) [32]. The 92 Pea Single Plant (PSP) accessions that were used for association mapping are listed in Additional file 1. The accessions were developed by selecting seeds from a single plant and were deposited with the USDA-ARS for inclusion in their PSP collection (https://npgsweb.ars-grin.gov/gringlobal/method.aspx?id=494267). All the PSP accessions are from the USDA Western Regional Plant Introduction Station and are freely available from the USDA (https://npgsweb.ars-grin.gov/gringlobal/search.aspx) under the Standard Material Transfer Agreement under the International Treaty on Plant Genetic Resources for Food and Agriculture, Convention on Biological Diversity. In addition, pea cultivars ‘Sonata’ (Dave Goulden, Plant & Food Research, Christchurch, New Zealand), ‘Primo’ (Cebeco, Lelystadt, The Netherlands) and breeding line SuperGreen (courtesy of Adrian Russell, Plant Research (NZ) Ltd., Lincoln, New Zealand) were grown as standards; and OSU442–15 (442–15) [33] was grown as a check line.

For starch extractions, peas were grown in two trials, a glasshouse trial held in 2010–2011 (GH2010) and a field trial held over the New Zealand summer of 2011–2012 (Field2011). No specific permissions were required for these trials which were conducted in accordance with local and national regulations. The GH2010 trial included 113 PSP lines, ‘Primo’, ‘Sonata’ and SuperGreen, grown two plants/pot in two pots; and 442–15 as the check, grown two plants/pot in eight pots. The trial consisted of two arrays of six tables with 20 pots/table, a total of 240 pots. Each array contained a complete replicate of lines, and these were positioned using a design derived from a block design, with blocks of 20 pots (one table). The trial was also designed to facilitate analysis of any trends or variation that may have resulted from laboratory processes such as starch extraction and the FACE analyses. The GH2010 trial design was generated with CycDesigN (http://www.vsni.co.uk/software/cycdesign) using a randomized resolvable block design with blocks of six, randomized across replicates. Pots were sown in September 2010 and harvested in January 2011, over the New Zealand spring/summer period. Plants were grown in a glasshouse under natural light until mid-November 2010, when very light shade cloth (30–35% shade) was placed beneath the glasshouse roof to help to regulate glasshouse temperature. Plants were grown in a bark-sand mix containing slow release fertilizer (Osmocote® Exact Standard) and were side-dressed with Nitrophoska Blue Special (Ravensdown Fertilisers, NZ) twice. The Field2011 trial was carried out near Lincoln, Canterbury, New Zealand at approximately E172° 28′ x S43° 37′ and included 112 PSP lines, ‘Primo’, ‘Sonata’ and SuperGreen with 442–15 grown as the check. Two replicate plots (10 seeds per plot) of the 114 test lines, two replicates each of ‘Primo’ and ‘Sonata’, four replicates of SuperGreen, and 34 plots of 442–15 were laid out in two adjacent blocks of 19 by 7 plots (a total of 266 plots), separated by a tractor track. Each block contained a complete replicate of the PSP lines plus one ‘Sonata’, one ‘Primo’, two SuperGreen, and 17,442–15 lines. The positions of the lines were determined using DiGGer experimental design software (http://nswdpibiom.org/austatgen/software/), with blocks of 19 × 7, 5 × 14, 19 × 1 and 1 × 14 with no autocorrelation. Plots were single row plots of 1.2 × 1 m. Standard cultural practices for pea were practiced and irrigation was applied to avoid water deficit.

### Candidate gene sequence selection

Twenty-five candidate genes (Table 1) from carbohydrate and starch metabolic pathways were selected based on a published pea starch biosynthetic pathway [15] and on the KEGG (www.genome.jp/kegg/pathway.html) Starch and Sucrose Metabolism pathway for *Arabidopsis thaliana*. For 13 of the genes, *P. sativum* cDNA sequences were directly available from GenBank. For nine of the remaining 12 candidate genes, the pea homologs of genes from other plant species were found as follows. First the relevant genes from *A. thaliana* were identified based on the KEGG Starch and Sucrose Metabolism pathway. Then the tBLASTx algorithm [34] was used to identify homologous pea sequences from among a database of 13,336 cDNA sequences obtained in our laboratory from Roche 454 sequencing of non-normalized cDNA libraries from developing pea seeds (cultivar ‘Primo’; 5, 15 and 25 days after pollination) (Genbank BioProject PRJNA288408). Three additional candidate gene sequences (phosphoglucan water dikinase, PWD; chloroplastic pullulanase 1, PUL1; and invertase inhibitor, InvInh) that were not previously characterized in pea, and also were not represented in the KEGG *A. thaliana* Sucrose and Starch Metabolism pathway diagram, were identified from the literature on starch metabolism in other plant species and then pea homologs were found among our cDNA sequence database using tBLASTx. Searches of the in-house database were carried out using Geneious Pro version 5.5.6 created by BioMatters (www.geneious.com).

**Table 1:** Pea carbohydrate metabolism candidate genes, primer sequences for fragment amplification and fragment characterisation

Gene, EC number, (GenBank accession, *locus*), *Genus and species*	Primer(s)	Primer sequences (5′ to 3′ direction)	PopSet alignment^a^ (location on accession)	Alignment length (bp)
Hexokinase, EC 2.7.1.1 (XM_003630659), *Medicago truncatula*	Ps_2048 (S), Ps_2049	F: CGGTTTTACGTTCTCGTTCC R: ATCTGCCTCCAGCCAATGT	694,184,588 (1003–1079)	291
Hexokinase, EC 2.7.1.1 (XM_003630659), *M. truncatula*	Ps_2050 (S), Ps_2051	F: AAGCGGAGTTTTTCGGAGAT R: ACCGCGATAAGCAACAATG	694,183,180 (1446–1545)	244
Phosphoglucomutase (plastidial), EC 5.4.2.2 (AJ250770, *rug3*), *Pisum sativum*	Ps_1321 (S), Ps_1322 (S)	F: GTCAACGCCAGCCGTTTC R: GGGTGTTTCCGTAAATCTTGTC	694,186,924 (566–636, 637–658)	471
Phosphoglucomutase (plastidial), EC 5.4.2.2 (AJ250770, *rug3*), *P. sativum*	Ps_1325, Ps_1328 (S)	F: AGGGTCTTGCACGATCAATG R: GGCTTCTCTCTCCCTGTGAA	694,182,794 (1651–1674, 1675–1752, 1753–1834)	374
Sucrose synthase, EC 2.4.1.13 (AJ012080, *rug4*), *P. sativum*	Ps_0685 (S), Ps_0689	F: TGACTGATGGTGCATTTGGT R: CGTTGGCCACAAGTAGTTCC	694,182,614 (376–401, 402–594)	378
Second sucrose synthase, EC 2.4.1.13, (AJ001071, *Sus2*), *P. sativum*	Ps_0076 (S), Ps_0079	F: ATATGTTGCTCAGGGGAAAGG R: ATTAACACGGACATACTCCCAAAC	694,184,180 (297–337)	374
Sucrose phosphatase, EC 3.1.3.24 (AY651774), *M. sativa*	Mt_0208 (S), Mt_0211	F: GAACCAGAAATGGGACAAGG R: TGCCACTGCAGTAATTCCTCT	694,182,434 (289–373)	235
Sucrose phosphate synthase, EC 2.4.1.14 (Z56278), *Vicia faba*	Ps_1581, Ps_1583 (S)	F: ACAGGAAATAGAAGAACAGTGGCGCT R: AGGACGGCATTCTCCAAACGCT	694,186,708 (1292–1409, 1410–1442)	569
Cell wall invertase, EC 3.2.1.26 (AF063246, *bfruct1*), *P. sativum*	Ps_0276 (S), Ps_0280	F: TGATCCTCAACTTCTGTGTAGTC R: TGCTAATGTAGGATAAACTCTGG	694,183,574 (1466–1560, 1561–1602)	303
Invertase inhibitor, putative (XM_004508064), *Cicer arietinum*	Ps_2042 (S), Ps_2043	F: TTAAATGAACCCCCACCAGA R: TCCAGAAGCACTTTCCCATC	694,182,042 (162–556)	411
ADP glucose pyrophosphorylase L1, EC 2.7.7.27 (X96766, *rb*), *P. sativum*	Ps_0036 (S), Ps_0039	F: GGGAGCTGACTATTACCAAACTGA R: CTTGATACCTTCACACACTCAACC	694,185,424 (1507–1567, 1568–1730)	374
ADP glucose pyrophosphorylase S2, EC 2.7.7.27 (X96765), *P. sativum*)	Ps_0057 (S), Ps_0065 (S)	F: GGCTACTGGGAAGACATTGGTA R: GATTCTCGCGTTCTTGTCAAC	694,187,965 (1149–1269, 1270–1368)	559
UDP-glucose pyrophosphorylase, EC 2.7.7.9 (AF435969), *Amorpha fruticosa*	Ps_1557 (S), Ps_1562	F: AGTTGGAAATTCCTGATGGAGCCGT R: AAGAAGACAACCAGCAAGGCCTCA	694,187,311 (1529–1610)	201
UDP-glucose pyrophosphorylase, EC 2.7.7.9 (XM_003616133), *M. truncatula*	Ps_1556 (S), Ps_1561	F: CCGCTACCGCTACCAACCTCG R: GCAACCCATAGTTGTCCCCAAGCC	694,186,492 (254–303, 304–397)	507
α-1,4-glucan phosphorylase L, EC 2.4.1.1 (Z36880), *V. faba*	Ps_1495 (S), Ps_1499	F: AGCTGTTGCACACGATGTCCCC R: GCTCTGGGATGCACAAAGTTGGGT	694,186,062 (994–1095, 1096–1181)	491
Starch synthase II, EC 2.4.1.21 (X88790, *rug5*), *P. sativum*	Ps_1315 (S), Ps_1317 (S)	F: ACAGCATTCCTGGATTGGAA R: TTGCGAAATATTGGACTGTCA	1,206,484,033 (587–969)	521
Starch synthase II, EC 2.4.1.21 (X88790, *rug5*), *P. sativum*	Ps_1320 (S), Ps_1319 (S)	F: TTATCGCGATCATGGTTTGA R: TTGGTATTTGGCAGCAACAA	694,183,776 (1809–2299)	491
Granule bound starch synthase, EC 2.4.1.21 (X88789, *lam*), *P. sativum*	Ps_0251, Ps_0255 (S)	F: GGGTAGAAACGCCTTTTCAG R: CCTCCAGTACCTCGATTTGC	694,186,276 (1067–1191, 1192–1369)	389
Granule bound starch synthase Ib, EC 2.4.1.21 (AJ345045), *P. sativum*	Ps_0499 (S), Ps_0503	F: AGAAAAGTCCGCTTCTTCCA R: TTGGTCAGGGAGATTGAGAAG	694,187,140 (534–626, 627–690, 691–756)	546
Starch branching enzyme II, EC 2.4.1.18 (X80010), *P. sativum*	Ps_2070 (S), Ps_2071	F: AGATTTGCTGCTCCCTACGA R: AACTTTGGCCCACATCAAAG	694,185,214 (709–844)	244
Isoamylase, isoform 1, EC 3.2.1.68 (DQ092413), *P. sativum*	Ps_1512 (S), Ps_1516	F: AGGGGGAGTTTGTCAGTGCCTCA R: AGACCATGCCACTGCAGCCT	694,187,747 (1906–1959, 1960–2040, 2041–2105)	441
Isoamylase, isoform 2, EC 3.2.1.68 (DQ092414), *P. sativum*	Ps_0155, Ps_0152 (S)	F: GATCCTTATGTCAATAGGTCAGGTG R: CCTGAGGCTATCCAAAATCAAA	N/A ^b^ 1056–1135	142
Isoamylase, isoform 3, EC 3.2.1.68 (DQ092415), *P. sativum*	Ps_1479 (S), Ps_1483	F: TGCTTCCCACACCCCCAACA R: TCGTAGGACCACTCTCAAGTAGAGCTT	694,184,796 (2220–2509)	511
Pullulanase 1, chloroplastic-like, EC 3.2.1.41 (XM_004496070)*, C. arietinum*	Ps_1471, Ps_1472 (S)	F: TGGTGGGACACCCGTTGCTT R: TCCTGCATCTCTCAGCTACACCGA	694,182,256 (2170–2226)	291
Pullulanase 1, chloroplastic-like, EC 3.2.1.41 (XM_004496070)*, C. arietinum*	Ps_1469 (S), Ps_1473	F: ACACTGGACCATCGTTGGCTTATGG R: GCACTCGCATCAGATTTTCCTTGGC	694,184,384 (2657–2717, 2718–2817, 2818–2846)	360
Beta-amylase 1-like, EC 3.2.1.2 (XM_004503530), *C. arietinum*	Ps_1518 (S), Ps_1521	F: CTGTGCTGCGTGGGCGTTCT R: TGGCATGTTCCAAGAGCCACCC	694,185,636 (762–810, 811–909, 910–996)	481
Beta-amylase-like, EC 3.2.1.2 (XM_003593956), *M. truncatula*	Ps_1523 (S), Ps_1525	F: GCTGTTCATGCTGAACCGATCAGAG R: TCTTTGTAACACTGTCCCGACCGA	694,183,376 (436–717)	501
Beta-amylase-like, EC 3.2.1.2 (XM_003593956), *M. truncatula*	Ps_1475 (S), Ps_1476	F: GCGGTCCACACGATGTGCCT R: TTCATGTCTTACACTGCTTGCATGCTC	694,185,848 (1179–1699)	521
Beta-amylase like, EC 3.2.1.2 (XM_004513491), *C. arietinum*	Ps_1602 (S), Ps_1606	F: TGCTGCTGAACTCACTGCTGGA R: TGAATCCCAAGGGAACGGCACT	694,182,984 (1347–1596)	481
4-α glucanotransferase, EC 2.4.1.25 (XM_003602434), *M. truncatula*	Ps_1596 (S), Ps_1601	F: TGGGTTTGGAGGTGGTCCCG R: TTGAGCAACGGAAGCCAGCG	694,185,004 (1547–1611, 1612–1655)	311
Phosphoglucan water dikinase, chloroplastic-like, EC 2.7.9.5 (XM_004497365), *C. arietinum*	Ps_1575 (S), Ps_1578	F: GCTCTTCAACCCTTGCCGCTCA R: GCATGCCTATTGGGACGGTGGT	694,183,978 (912–956, 957–1040)	268
Phosphoglucan water dikinase, chloroplastic-like, EC 2.7.9.5 (XM_004497365), *C. arietinum*	Ps_1573 (S), Ps_1579	F: TCCAGCGCCAATGTGGAGGA R: AGGTTGGGGCCTTGTCTGAACA	694,187,529 (3119–3629)	511

^a^The GenBank PopSet alignment number

^b^Not applicable, the sequence is less than 200 bp therefore not accepted by GenBank

For each candidate gene studied, the EC number of the encoded enzyme and GenBank accession for the most similar mRNA sequence are indicated. The species related to that GenBank accession is also indicated. Genbank accessions were accessed on 9 August 2016. The primers that were used for resequencing are indicated (S). GenBank PopSet numbers for the pea candidate gene sequence fragment alignments are provided, along with alignment lengths

### Candidate gene resequencing and sequence analysis

Primer pairs for the pea candidate gene sequences were designed using Primer3 software (http://frodo.wi.mit.edu/primer3/). The default parameters for primer design were an optimal Tm of 60 °C and an optimal length of 20 nucleotides. Two to three sets of primers were designed for each sequence. When designing primers, attempts were made to place the primer binding sites so that they flanked intron sequences to improve the chances of identifying polymorphic sites. The possible locations of introns in the pea genomic sequences for the candidate genes were estimated by aligning the pea candidate gene cDNA sequences with pea or other legume genomic sequences using the BLASTx algorithm, or if legume genomic sequences were not available, then *A. thaliana* genomic sequences were used.

Total DNA was extracted from young leaves of pea lines as described by Timmerman et al. [35]. For a minority of the PSP lines, extracted DNA did not reliably amplify during PCR. For those lines, whole genome amplification was carried out to circumvent the problem, using the illustra GenomiPhi V2 DNA amplification kit (GE Healthcare Life Sciences) following the manufacturer’s instructions. The primer sequences used to identify polymorphisms are listed in Table 1. In a standard reaction, genomic DNA fragments were amplified in 15 μl containing 1× PCR buffer (various suppliers), 200 μM of each dNTP, 200 nM of each PCR primer, 0.3 U of Taq polymerase (various suppliers) and approximately 20 ng of total or genome amplified DNA. Mg^2+^ concentrations in PCR reactions were optimized where necessary.

PCR products were treated with exonuclease I and either shrimp or rAPiD alkaline phosphatase (Roche) [36] and then sequenced using BigDye ver. 3.1 (Applied Biosystems) and an ABI3130 Genetic Analyzer (Applied Biosystems). PCR primers used to prime the sequencing reactions are indicated in Table 1. Bases were called using either SeqScape ver. 2.1 (Applied Biosystems), ABI Sequence Analysis Software version 5.3, or Geneious Pro version 5.5.6 software. Alignments were constructed using ClustalX version 2.1 [37]. Polymorphisms were confirmed by visual inspection. Linkage disequilibrium among polymorphisms was calculated and plotted in R using the ‘genetics’ (ver 1.3.8.1) and ‘Ldheatmap’ packages (ver 0.99–1) [38]. For the nucleotide polymorphisms associated with CLD variation and which would generate amino acid substitutions, estimation of the likelihood that a variant might have an effect on protein biological function was carried out using the Protein Variant Effect Analyzer (PROVEAN) ver. 1.1 [39]. Sequences are lodged as population set (PopSet) alignments (Table 1) with GenBank (sequences KM360195-KM360301, KM510517-KM513542 and KY983278-KY983354).

### Starch extraction

Starch was extracted from the GH2010 and Field2011 samples based on the method described by Takeda et al. [40]. A subsample of dried pea seed (5 g) harvested from each pot or plot was soaked in 30 ml 0.2% NaOH for 2 days at 4 °C. An additional 40 ml of 0.2% NaOH was added and the soaked peas were blended for 3 × 20 s bursts using a household stick blender (200 W) then centrifuged at 3200 g for 20 min. The pellet was resuspended in 20 ml of 0.2% NaOH then sieved through three sieves with mesh sizes of 420 μm, 100 μm and 75 μm, with additional 0.2% NaOH used to ensure a good starch recovery, up to a maximum volume of 45 ml. Starch was pelleted by centrifugation of the filtrate at 3200 g for 20 min. The supernatant was discarded and any layer of non-starch material on the top of the starch pellet was removed with gentle scraping using a stainless steel spatula. The resulting starch pellet was resuspended in 40 ml water and 0.5 ml of 0.5 M MOPS to neutralize the suspension then centrifuged at 3200 g for 30 min, the supernatant discarded and any non-starch layer above the starch pellet removed by gentle scraping. The pellet was then washed three times with 40 ml water with centrifugation at 3200 g for 10 min. The final starch pellet was dried at 37 °C for 24 h, broken up with a mortar and pestle, sieved and stored at ambient temperature.

### Fluorophore-assisted carbohydrate electrophoresis and data analysis

The CLD of debranched starch from each sample was estimated using FACE as described by Murray et al. [27]. For debranching and labelling of the extracted pea starch, the scaled-down protocol was followed. Debranching was carried out using 10 ± 0.5 mg starch. Labelling was carried out on a subsample of 103 μg of debranched starch per labelling reaction using 8-amino-1,3,6-pyrenetrisulfonic acid (APTS) fluorophore at 20 μg/μl. The FACE labelling reactions were diluted with water before electrophoresis to ensure that the fluorescence signal of the tallest peak fell between 7500 and 2000 relative fluorescent units (RFU) when electrophoresed on an ABI3130 Genetic Analyzer (Applied Biosystems). All analysis was done using GeneMarker software versions 1.85 and 2.2 (SoftGenetics, State College, PA, USA; www.softgenetics.com) as described by Murray et al. [27]. For each sample, degrees of polymerization (DP) between 6 and 40 were considered. Peak areas for these DPs were exported from GeneMarker, and then converted to relative abundances, expressed as molar proportions of the total peak area for that sample. For each sample, the sum of the peak area molar proportions was equal to 1.

CLD data were analysed as described by Murray et al. [27]. Briefly, a standard Poisson log-linear model for the analysis of contingency Tables [41] was used to analyse the table of samples by DP molar proportions. This approach was taken since numbers of fluorescently labelled starch chains of each DP underlie the molar proportions. To adjust for the data being proportions rather than counts, the dispersion was estimated rather than fixed at 1 (the expected value for counts). The main aim of this analysis was to explore whether there were any substantial differences in the DP distributions between lines. These effects were assessed with F-tests within the analyses of deviance that were done. Correspondence analysis [42] was used to explore patterns in the contingency tables. The results are presented as asymmetric biplots, with DP as standard coordinates where the plot is a projection of DP when treated as axes in multidimensional space (26 dimensions). Analyses were carried out in GenStat 14th edition [43].

### Percent amylose estimation and data analysis

The amylose content of the pea starch samples was determined using an iodine binding assay optimized for measurement in a 96 well plate [44], with the following modifications. Pea starch samples, in 50 ml Falcon tubes, were placed in a Labconco Centrivap Concentrator (Kansas City, MO 64132, USA) and re-dried under vacuum for 2 h at ambient temperature to remove any residual moisture, before a 5 mg subsample was weighed for analysis of %amylose. The starch was dispersed in 1 ml of 90% DMSO in water by heating to 95 °C for 60 min with vortexing every 10 min, then cooled for 5 min. A 100 μl aliquot from each sample was pipetted into a 0.5 ml microfuge tube, 100 μl of I_2_ solution (3.04 g I_2_/L in 90% DMSO) was added and the tube vortexed for 30 s. The tubes were incubated at room temperature for 30 min before 20 μl was aliquoted in quadruplicate into a 96 well clear, flat bottomed polystyrene plate (Greiner Bio-One 655,101) and 180 μl of deionized water added to each well. The plate was shaken for 30 s before reading the absorbance. A set of ten standards containing 0, 5, 10, 15, 20, 25, 30, 50, 75 and 100% amylose was also added to each plate in triplicate. The standards were made up using amylose, Type III from potato, and amylopectin from potato (A0512 and A8515, respectively, Sigma-Aldrich, St Louis, MO, USA). Absorbance of each well was measured at 620 and 510 nm in a SpectraMax M2 platereader using SoftMax® Pro 5 software.

Calibration curves for each plate were developed by plotting the Abs_620nm_ – Abs_510nm_ versus the % amylose of the calibration standards. Exploration of these curves showed clearly that the relationships were non-linear, justifying the inclusion of the quadratic term in the calibration regressions, where.

Abs_620nm_ – Abs_510nm_ = c + b(%amylose) + a(%amylose^2^).

(a = quadratic term, b = linear term, c = constant term). The resulting calibration curves all fitted very well (R^2^ > 99.65) with the lack of fit between the means for the standards and each plate and the fitted curve being minor (<0.1% of the total variation across all plates). Parameters a, b and c were moderately variable between the plates with % CVs (standard deviation as % of mean) of 10.9, 6.5 and 9.0%, respectively. The parameters of the above equation were used to convert the Abs_620nm_ – Abs_510nm_ (AbsDiff) into estimated %amylose for each well using the following equation:

$$ \mathrm{Estimated}\%\mathrm{Amylose}=\frac{\hbox{-} \mathrm{b}+\sqrt{{\mathrm{b}}^2\hbox{-} 4\mathrm{a}\left(\mathrm{c}\hbox{-} \mathrm{AbsDiff}\right)}}{2\mathrm{a}} $$

To obtain pot (GH2010) or plot (Field2011) means, data from the two trials were analysed separately. The estimated % amylose for each well was analysed using methods appropriate for percentage data. Since there were up to four wells per pot or plot (some odd data were excluded), starch extractions from replicate pots or plots for most lines, potential spatial effects from the trials, and effects relating to the plates not corrected for by the calibration regressions, an initial analysis was carried out to assess the importance of each of these. A hierarchical generalized linear modelling approach [45] was used. In this, fixed effects (lines; round vs wrinkled seeded) were fitted with a binomial distribution with a logit link and dispersion estimated. Random effects (pot (GH2010) / plots (Field2011); plates; other spatial factors) were fitted as random effects with a beta distribution and logit links. The random effects were assessed with a *Χ* ^2^-test of the change in likelihood on dropping a term, as implemented in GenStat’s HGRTEST procedure [46]. Only important random terms were included in the final analysis. Fixed effects were assessed similarly to random effects, using GenStat’s HGFTEST procedure. Mean %amylose values were obtained as predictions on the link (logit) scale, and back-transformed for presentation. All data manipulation and analyses were carried out with GenStat [47].

### Association mapping analysis

Association mapping was carried out using 92 PSP lines (Additional file 1). Population structure was estimated on the basis of polymorphisms at 55 background markers, consisting of 13 SCAR markers, 12 SSR markers and 30 RAPD markers. These markers revealed 140 polymorphisms. Two approaches were used. In the first, the Bayesian, model-based approach implemented in STRUCTURE version 2.3.4 software [30] was used to determine the number of sub-populations which best represented the data, based on a no-admixture model and uncorrelated allele frequencies, and then to assign lines to subpopulations. The software was run to test from one to eight subpopulations (*K*) with five replicates, a burn-in period of 100,000 and then 500,000 replicates. The STRUCTURE analysis was carried out on the full set of 92 PSP lines and on a subset of 83 PSP lines that had the round seed phenotype (*RR* genotype at the *r* locus). The most likely number of subpopulations (*K*) was determined by plotting the Ln probability of the data (Ln P(D)) versus *K*. The resulting Q matrix for the most likely number of subpopulations was used for association mapping. In the second approach, a PCA of both the *n* = 92 and *n* = 83 sets of PSP lines × 55 background loci datasets was carried out using Genstat 14th ed. [43], producing a P matrix. Kinship (K) matrices were calculated for both the *n* = 92 and *n* = 83 PSP lines datasets, with the results rescaled to between 0 and 2, using the same polymorphism data, using TASSEL version 3.0.165 [31, 48].

Association mapping was carried out using the mixed linear model (MLM) function implemented in TASSEL [48]. To determine the best model to use for association mapping, the MLM + Q + K and MLM + P + K models were analysed using the 92 PSP lines dataset of 280 polymorphisms from 25 candidate genes, phenotypic variation at the *r* locus (round versus wrinkled seeds), and trait values that included %amylose content and CLD presented as mean peak area proportions from DP6 to DP40 for starch extracted from 92 PSP lines from the GH2010 and Field2011 trials. In the MLM analyses, the marker effects and P or Q matrices were fitted as fixed effects and the kinship matrix (K) and residual were fitted as random effects. Polymorphisms (SNPs and indels) in sequences from 25 starch and carbohydrate metabolic genes were extracted from sequence fragment alignments using TASSEL, with a required minimum minor allele frequency of 5%. For the MLM analysis, variance components were re-estimated after each marker and the compression level was optimized during the analysis. To determine which of the two models gave the best fit, quantile-quantile plots were drawn for the -log_10_ of the 281 raw *p*-values obtained for the traits which gave significant marker-trait associations. For the *n* = 83 round lines, association mapping was carried out using the MLM + Q + K model. Q-values [49] were calculated to provide measures of the significance of the association tests obtained from the MLM by estimating the minimum false discovery rate (FDR) that occurs if that test is called significant. Q-values were calculated using the QVALUE software [49] implemented in R. The false discovery rate (FDR) was set to 0.05 (*n* = 92 lines) or 0.10 (*n* = 83 lines), and the bootstrap method was used to estimate π_0_, the overall proportion of true null hypotheses. Characteristics of the distributions for significant associations were visualized using beanplots [50].

## Results

### Variation in percent amylose and starch chain length distribution

In the GH2010 and Field2011 trials, 116 and 115 pea lines respectively were grown, as well as OSU442–15 which was incorporated as a check line. Of these lines, there were 92 that yielded adequate amounts of seed from both trials for starch extraction, analysis of % amylose and FACE analysis.

From the GH2010 and Field2011 trials, the mean %amylose in starch from the wrinkled (*rr*) seeded lines was 63.3% (range 59.2 to 69.7) and 62.1% (range 56.7 to 69.3), respectively; while for the round (*RR*) seeded lines the mean %amylose was 37.2% (range 27.6 to 44.1) and 38.2% (range 34.5 to 44.6), respectively. From the GH2010 trial, most of the differences between lines were related to pea type (round versus wrinkled seeded, *p* < 0.001) with little difference between the round lines (*p* = 0.821) and relatively more between the wrinkled lines (*p* = 0.038). From the Field2011 trial, most of the major differences were again associated with the pea type (*p* < 0.001) but there were more notable differences between lines within the two types (*p* = 0.014 for round lines; *p* < 0.001 for wrinkled lines). The greater significance of the differences in terms of *p*-values for the %amylose values for lines from the Field2011 trial occurred because there was more consistency between replicate plots of the Field2011 trial than there had been for replicate pots of the GH2010 trial. The mean %amylose and associated 95% confidence intervals for each of the 92 lines used for associaton mapping from the GH2010 and Field2011 trials are presented in Additional file 1.

**Fig. 1:**
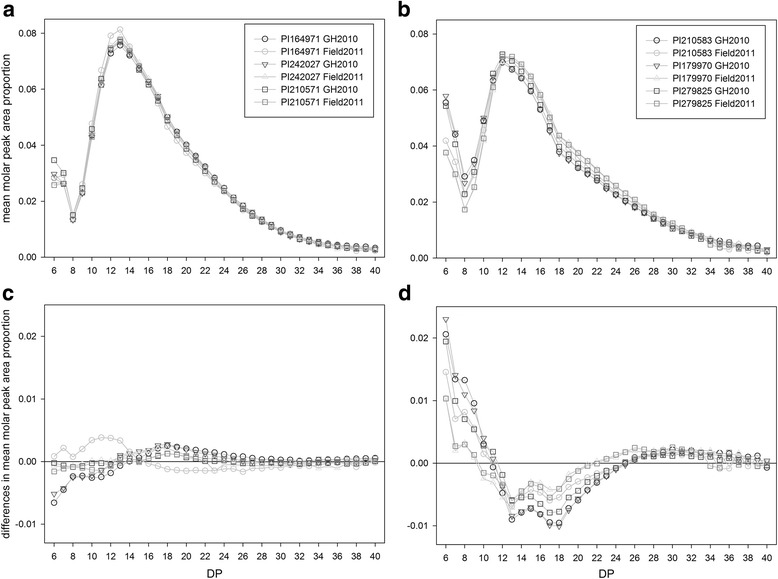
Mean chain length distributions (CLDs) for debranched pea starch from three round and three wrinkled pea single plant (PSP) lines grown in the GH2010 and Field2011 trials. Profiles showing mean molar peak area proportions for starch from pea lines having: **a** the round (*RR*) and **b** the wrinkled seeded (*rr*) genotype at the *r* locus; and difference plots for **c** starch extracted from round seeded lines and **d** wrinkled seeded lines. Difference plots show each sample’s mean molar peak area distribution minus the mean molar peak area distribution for the 92 PSP lines

Starch was characterized for CLD by carrying out FACE on debranched starch extracted from pea seeds. Typical CLD profiles for debranched pea starch are shown in Fig. 1. Profiles are shown from both the GH2010 and Field2011 trials for six PSP lines; three each that have round (A and C) and wrinkled (B and D) seed phenotypes. The profiles have been graphed as the mean molar peak area proportion versus DP (A and B); and as difference plots, in which the value plotted is the mean molar peak area proportion for the PSP line under consideration minus the mean total molar peak area proportion for all 92 PSP lines versus DP (C and D). These plots show the general trends associated with *r* locus phenotype. In wrinkled seeded lines the relative abundance of DP6 to DP12 chains was greater than in round lines and concomitantly the relative abundance of DP13 to DP24 chains was less in wrinkled than in round seeded lines. Analysis of the CLD resulting from the FACE using a standard Poisson log-linear model for the analysis of contingency tables found that overall the DP distributions varied significantly (*p* < 0.001) between lines.

**Fig. 2:**
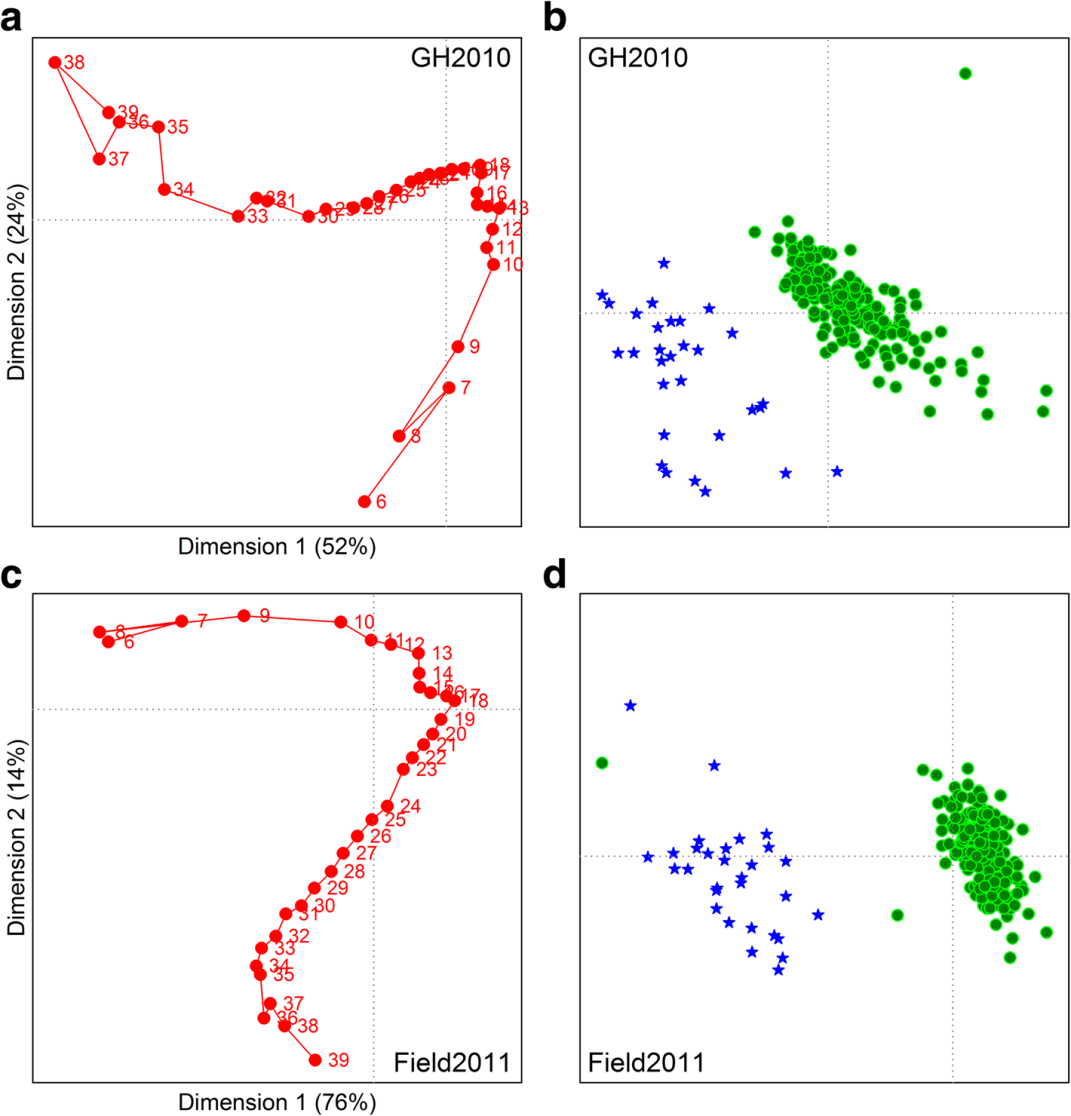
Correspondence analysis bi-plots for debranched pea starch from the GH2010 (**a** and **b**) and Field2011 (**c** and **d**) trials, derived from a two-way contingency table with the variables DP (columns) and pea lines (rows), and plotted against the first and second dimensions. Degrees of polymerization (DP) are plotted in graphs **a** and **c** *(red circles*) while the pea lines in each pot (GH2010) or plot (Field2011), including controls, are plotted in graphs **b** and **d** (round seeded lines are indicated with green circles, wrinkled seeded lines with blue stars). **a** and **c** versus **b** and **d** are not plotted on the same scale since the points for the pea lines (**b** and **d**) occur near the centroid of plots A and C and occupy only a small area of the DP points

Correspondence analysis was employed to assist with identifying differences between the CLD distributions obtained from the two trials (Fig. 2). CLD distributions from all the lines that produced sufficient seed for FACE analysis were included in these analyses of each trial (110 PSP lines plus Primo, Sonata, OSU442–15 and Supergreen for GH2010; 112 PSP lines plus Primo, Sonata, OSU442–15 and Supergreen for Field2011). For the GH2010 trial, the first two components accounted for 76% of the CLD variation, with the first component accounting for 52%. Therefore, the two-dimensional plot shows many of the important patterns in the data. However, 24% of the variation was not associated with the first two components. Nine components were needed to explain 95% of the variation. The greatest differences between the distributions were associated with the highest and lowest DP. The first dimension separated the highest DPs (DP36 to DP39) from lower DP scores (DP10 to DP18) and also separated the wrinkled from the round seeded lines. The second dimension separated the low DP (≤9) from the rest of the distribution and showed some effect of the wrinkled versus round seeded lines. For the Field2011 trial, the first two components accounted for 90% of the variation in the data, with the first component accounting for 76%. Therefore, this two-dimensional plot captures most of the important patterns in the data. For the CLD plot distributions, the greatest differences were associated with the DP values ranging from DP6 to DP18 in dimension 1, and in dimension 2 with the low (DP6 to DP10) to the high (DP39) DP values. For the analysis of pea lines, the obvious difference is the clear separation between wrinkled and round seeded lines, mostly associated with dimension 1 and seen in both trials.

Correlations between the CLD mean peak area proportions from DP6 to DP40 for the starch extracted from the PSP lines grown in the GH2010 versus Field2011 trials are presented in heat maps (Additional file 2) comparing all 92 lines (round and wrinkled, top panel) and the 83 round lines only (bottom panel). Strong correlations, both positive and negative, (range − 0.879 to 0.921) were observed when all lines were considered, no doubt because of the major effect of *r* locus on CLD distributions. Weaker correlations (range − 0.365 to 0.492) were seen when the mean peak area proportions at all DP values from the round lines only were compared for the GH2010 versus Field2011 trials.

### Candidate gene selection, genotyping and linkage disequilibrium

**Fig. 3:**
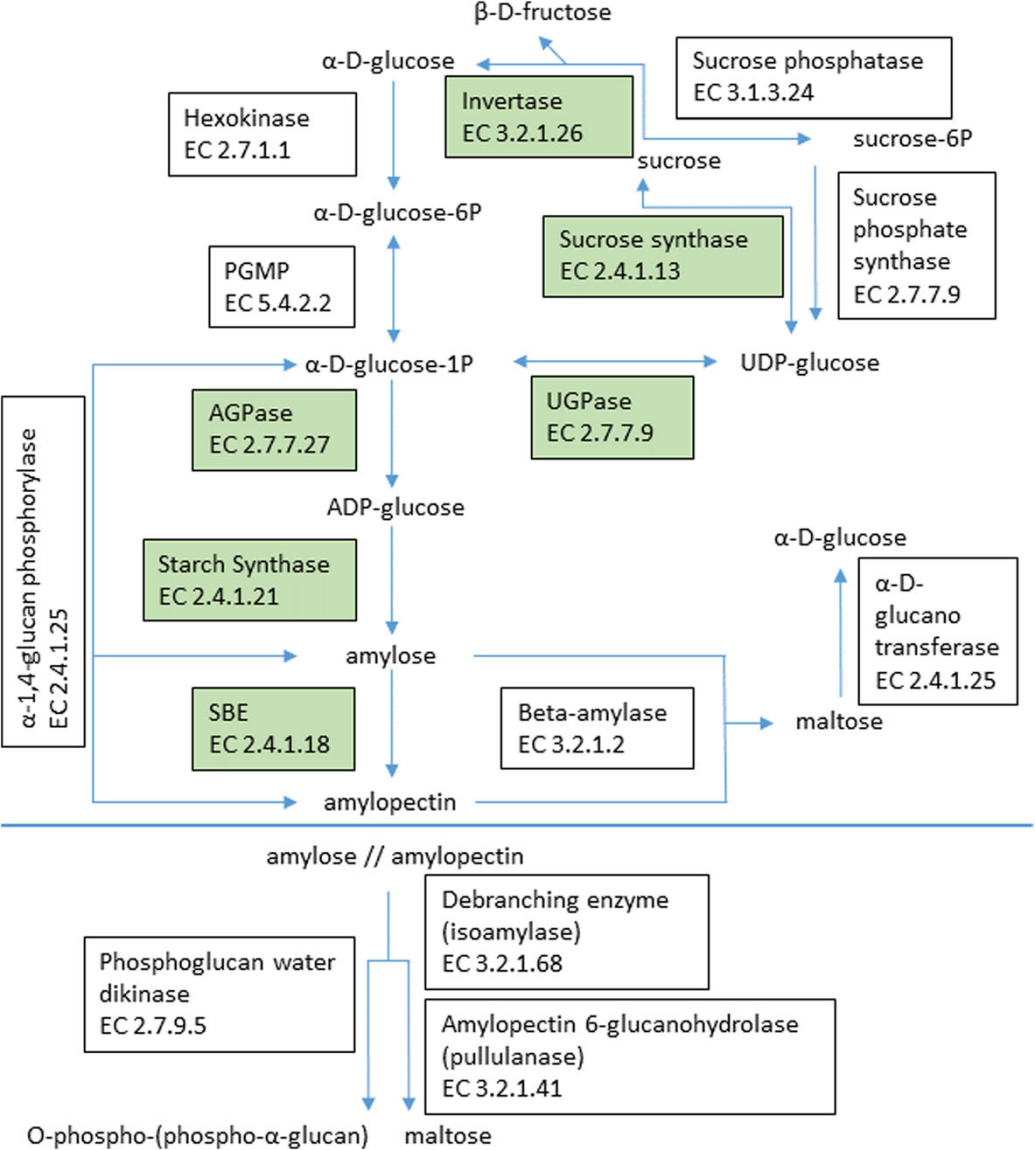
A partial carbohydrate and starch metabolic pathway showing the 16 enzyme-catalyzed reaction classes for which polymorphisms in 25 candidate gene sequences and *r* locus were characterized. Enzyme classes where significant association between candidate gene polymorphism and CLD were identified are shown in *shaded boxes*. Enzyme Commission (EC) numbers are shown

Partial sequences of 25 pea candidate carbohydrate and starch metabolism genes were assessed in this study (Table 1) giving a total of 280 polymorphisms with minor allele frequencies ≥5%. The *r* locus phenotype (round versus wrinkled seed) was treated as the 281st polymorphism, and although the phenotypes were scored, these were treated as genotypes since the pea lines being used are inbred and segregation of the round phenotype (*RR* or *R*_ genotype) was never observed in the harvested seeds. These candidate gene sequences and *r* locus represent 16 enzyme catalysed reaction classes in the pea carbohydrate and starch metabolic pathways (Fig. 3), including activities involved in precursor supply, chain elongation, chain branching, debranching, and phosphorylation and degradation. More than one candidate gene sequence was characterized for six of the enzyme classes. These were: AGPase (*AGPS2*, *AGPL1*), starch synthase (*StSynII*, *GBSSI*, *GBSSIb*), isoamylase (*ISA1*, *ISA2*, *ISA3*), sucrose synthase (*SuSy*, *SecSuSy*), invertase (*CWI*, *InvInh*) and beta-amylase-like (BAM-like, three beta-amylase-like candidate gene sequences). For some candidate genes, two genomic fragments were resequenced: *PGMP*, *StSynII*, *PUL1*, hexokinase (*Hex*), phosphoglucan water dikinase (*PWD*), UDP-glucose pyrophosphorylase (*UGPase*), and a BAM-like sequence. The candidate gene sequences for this study were either previously characterized pea genes (*n* = 13) involved in carbohydrate or starch metabolism or homologs of genes involved in carbohydrate or starch metabolism in other plant species (*n* = 12).

Linkage disequilibrium (LD) was analysed between all pairs of segregating sites with a minor allele frequency ≥ 5%, including the *r* locus phenotype, with the primary aim of understanding the chance of a correlation between polymorphisms in different candidate genes, and the secondary aim of understanding LD decay within pea genes and how this might influence the ability to detect association between traits and polymorphisms. A heat map is presented in Additional file 3 showing LD (r^2^) between the polymorphisms in the 25 candidate genes and *r*. The strong LD within sequences from a single gene is clearly observed, while in general only weak or no LD was observed between the sequences from different carbohydrate or starch metabolism genes. The strongest LD observed between different candidate gene sequences was r^2^ = 0.347, observed for polymorphisms in *InvInh* and *Iso1*. The ability to understand LD decay within these pea genes using these data is limited because of the short length (≤ 569 bp) of the alignments. However, since two fragments from different regions of their respective genes were sequenced for seven of the pea candidate genes (*Hex*, *PGMP*, *UGPase, StSynII*, *Pul1*, *BAM-1523-1475* and *PWD*), there was the opportunity to examine the extent of LD in different parts of a single gene. The maximum LD (r^2^) that was observed between polymorphisms within a fragment was 1.0, while the maximum LD observed between polymorphisms in different fragments of the same gene ranged from 0.340 (*Hex*) to 0.833 (*UGPase*). This analysis provides only limited information on LD decay in this panel of pea lines since the whole genes were not sequenced and therefore the physical distances between sites are not known.

### Population structure

Two approaches, the model-based Bayesian software STRUCTURE and PCA, were taken to estimate population structure in the PSP pea lines considered in this study, based on 140 polymorphisms obtained from 55 background molecular markers. These analyses were applied to the full set of 92 PSP pea lines (containing both round and wrinkled seed types) and the subset comprising the 83 lines with the round seed phenotype.

**Fig. 4:**
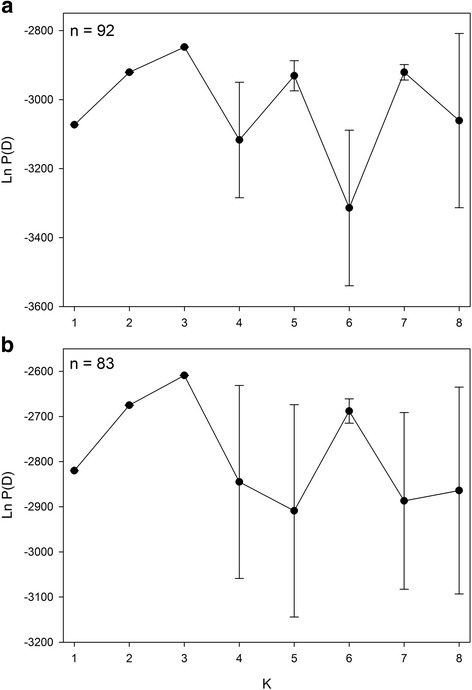
Population structure estimation using STRUCTURE. Plots of the estimated Log probability of the data (Ln P(D)), averaged over 7 replicates, for between 1 and 8 subpopulations (K) obtained from analysis of 55 background markers in STRUCTURE of the *n* = 92 (**a**) and *n* = 83 (**b**) PSP lines. Error bars show standard deviations

Model-based population structure estimation using STRUCTURE with the *n* = 92 and *n* = 83 datasets distinguished three (*K* = 3) likely subpopulations. Plots for between one and eight subpopulations (K) showed that the estimated Log probability of the data (Ln P(D)), averaged over 7 replicates, peaked at *K* = 3, indicating that three subpopulations was the best estimate for both the *n* = 92 and *n* = 83 association panels (Fig. 4a and b). Subpopulation membership of individual lines is indicated in Additional file 1. The *K* = 3 Q-matrices were used in association mapping.

In the PCA of the *n* = 92 dataset, the first eight principal components accounted for >98% of the variation, with PC-1 accounting for 54.7% and PC-2 for 16.6%. The major inflection points in the scree plots obtained from PCA indicated three subpopulations. In addition, examination of a PCA biplot of the first two PCs shows that the PSP lines fall into three subpopulations when PC-1 is considered. Consequently, the *P* = 3 matrices were used in association mapping. PCA and scree plots for the *n* = 92 dataset are shown in Additional file 4.

### Association analysis

A total of 280 polymorphisms in 25 carbohydrate and starch metabolism candidate genes, with the *r* locus phenotype treated as the 281st polymorphism, were tested for association with 35 CLD traits consisting of the mean molar peak area proportions at DP6 through DP40 as well as with percent amylose content of the extracted starch from the GH2010 and Field2011 trials. The association mapping analysis was carried out using the MLM + Q + K, MLM + P + K models. Quantile-quantile (Q-Q) plots of the -log_10_(P) were drawn for the DP which gave the most significant associations (Additional file 5) to identify the best association mapping model. The Q-Q plots indicated that the MLM + Q + K model, which controls both for population structure and underlying familial relatedness, provided a similar or better overall fit than the MLM + P + K model to the expected *p*-values for all traits, assuming a normal distribution for *p*-values. Therefore, association between candidate gene polymorphisms and CLD is reported based on the MLM + Q + K model. Associations were considered as significant when they met the α < 0.05 (for *n* = 92 lines) or α < 0.10 (for *n* = 83 lines) criteria for minimising FDR based on Q-values. Suggestive associations that have low *p*-values (*p* < 0.01) or that meet the α < 0.10 criterion are also discussed.

Since the *r* locus phenotype had an obvious effect on the CLD pattern of debranched isolated starch (Fig. 1), association mapping was carried out on all 92 lines that were fully phenotyped and separately on the subset of 83 round seeded lines. When all 92 lines were considered, polymorphisms in seven of the 26 genes/loci were found to be significantly associated with CLD phenotypes (Table 2). Of these seven candidate genes, three of the genes/loci were identified from both the GH2010 and Field2011 environments: *r* locus, *UGPase*, and *AGPS2*. The polymorphisms in the seven genes/loci were associated with CLD at DP10, DP16, DP17, DP34, and DP39 in the GH2010 environment; and with DP17, DP18 and DP23 in the Field2011 environment. The *r* locus had the major effect, explaining 83% of the variation at DP17 from the GH2010 trial and 89% of the variation at DP18 from the Field2011 trial. SNPs in *AGPS2* and *UGPase* were associated with CLD variation from both trials and explained from 14.3% (*AGPS2*, GH2010) to 22.2% (*UGPase*, Field2011) of the variation at the DPs with which they were most strongly associated. For four other loci, association with CLD was detected from only a single environment each. These were: *CWI* and *SecSuSy* associated with DP39 and DP29, respectively, from the GH2010 trial; and ADP glucose pyrophosphorylase L1 subunit (*AGPL1*, *rb*) and *StSynII* (*rug5*) associated with DP23 and DP17, respectively, from the Field2011 trial. Suggestive associations involving an indel polymorphism in *PWD* at site 49 of the alignment were detected from both the GH2010 (DP33, *p* = 6.76 E-04, q-value = 0.081) and Field2011 (DP 9, *p* = 1.23 E-03, q-value = 0.158) trials. Some of the associations of interest involved more than one polymorphic site within a sequenced candidate gene fragment (Table 2). These sites were in strong or complete linkage disequilibrium (Additional file 4).

**Table 2:** Summary of the associations between candidate carbohydrate metabolism gene sequences and starch chain length distribution (CLD) peak areas

Environment Glasshouse 2010. All PSP lines, *n* = 92.						
Gene	Sequence alignment ^a^	Site(s) on alignment ^b^	CLD peak with lowest *p*-value	*p*-value	R^2^ (%)	Q-value
Starch branching enzyme I (*r* locus)	n/a ^c^	n/a	DP17	7.50 E-39	83.4	1.92 E-36
Cell wall invertase	694,183,574	24 (T/G)	DP39	1.23 E-04	17.3	0.010
ADP glucose pyrophosphorylase S2 subunit	694,187,965	237 (indel)	DP10	1.83 E-04	14.3	9.13E-03
UDP glucose pyrophosphorylase	694,186,492	18 (T/G), 25 (C/G), 56 (T/G), 274 (T/G)	DP16	8.50 E-04	12.3	0.039
Second sucrose synthase	694,184,180	148 (C/T), 299 (indel)	DP34	1.52 E-03	11.8	0.015
Environment Glasshouse 2010. Round seeded PSP lines, *n* = 83.						
Second sucrose synthase	694,184,180	128 (T/A), 134 (T/indel/C)	DP29	2.68E-04	16.9	0.027
Invertase inhibitor	694,182,042	357 (T/C)	DP18	3.07 E-04	15.6	0.086
ADP glucose pyrophosphorylase S2 subunit	694,187,965	517 (G/T)	DP10	1.16 E-03	12.4	0.062
Environment Field 2011. All PSP lines, *n* = 92.						
Starch branching enzyme I (*r* locus)	n/a	n/a	DP18	8.81 E-53	88.6	1.72 E-50
UDP glucose pyrophosphorylase	694,186,492	25 (C/G), 56 (T/G), 274 (T/G)	DP17	2.62 E-06	22.2	1.02 E-04
ADP glucose pyrophosphorylase S2 subunit	694,187,965	72 (T/C)	DP23	6.58 E-04	12.1	0.027
ADP glucose pyrophosphorylase L1 subunit (*rb* locus)	694,185,424	9 (A/C)	DP23	8.94 E-04	12.1	0.027
Starch synthase II (*rug5* locus)	1,206,484,033	307 (G/A)	DP17	1.26 E-03	15.9	0.027

^a^GenBank PopSet identification number

^b^Where more than one site is shown in this column, they all had the same *p*-value; (major/minor) alleles are shown

^c^Not applicable. The *r* locus genotype was determined by recording the round or wrinkled seed shape, so the alignment and site information are not applicable

Associations were identified using the mixed linear model approach with adjustment for population structure using Q + K matrices, implemented in the Tassel package. For all the pea lines (*n* = 92), associations that meet the α < 0.05 criterion for minimising the false discovery rate (FDR) are shown, while for the round only pea lines (*n* = 83) associations that meet the α < 0.10 criterion are shown

**Fig. 5:**
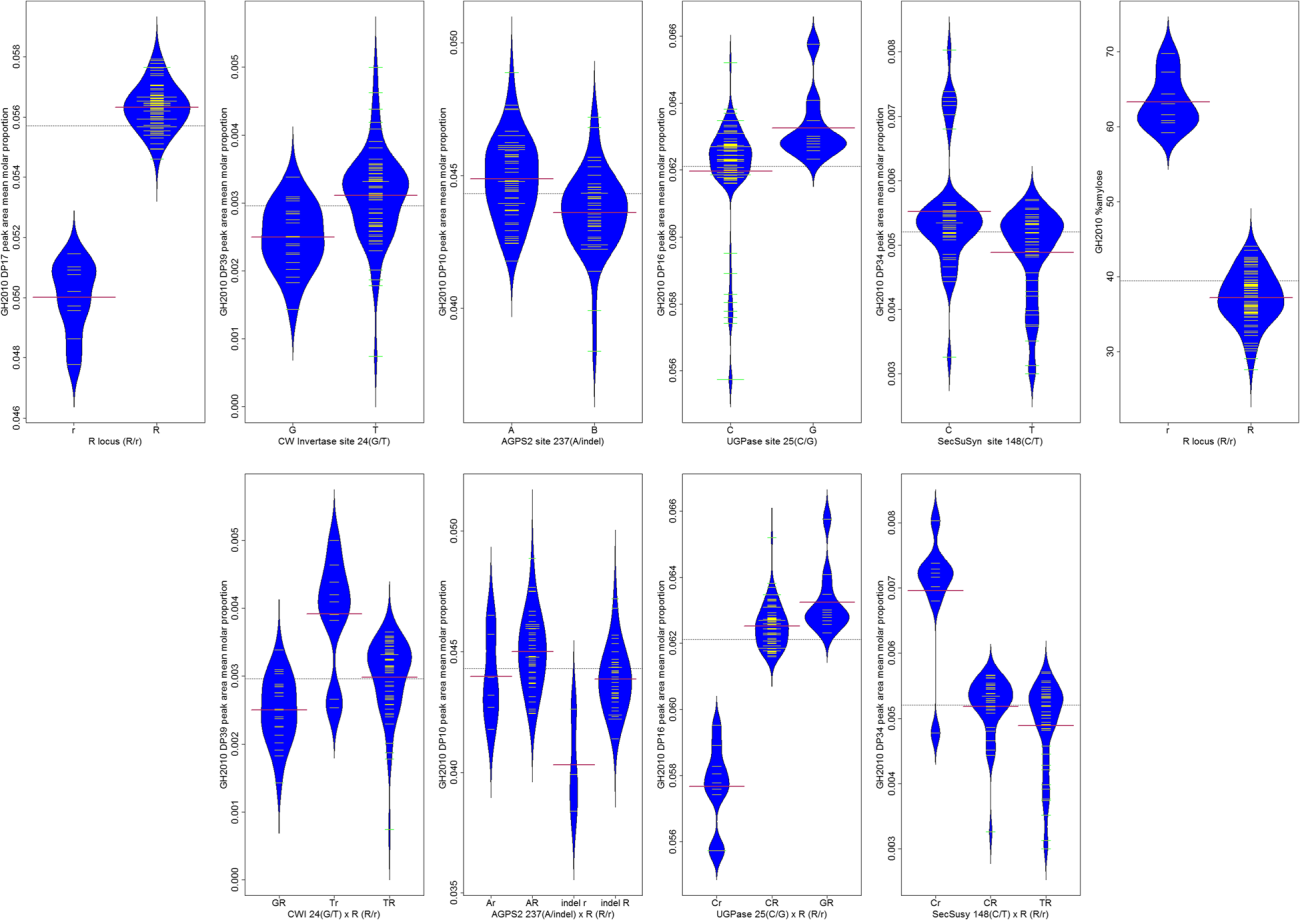
Beanplots showing the distributions of trait values from the GH2010 environment and *n* = 92 PSP lines for alleles of the associated locus-trait combinations. The means and distributions of the mean molar peak area proportion values are shown, grouped by allele, or by combinations of alleles from *r* and other loci. Allelic means (*red lines*) and individual PSP lines’ mean values (*yellow lines*) are shown. The second row contains plots showing pairwise interactions between alleles of *r* locus and the associated allele of the locus directly above each plot

**Fig. 6:**
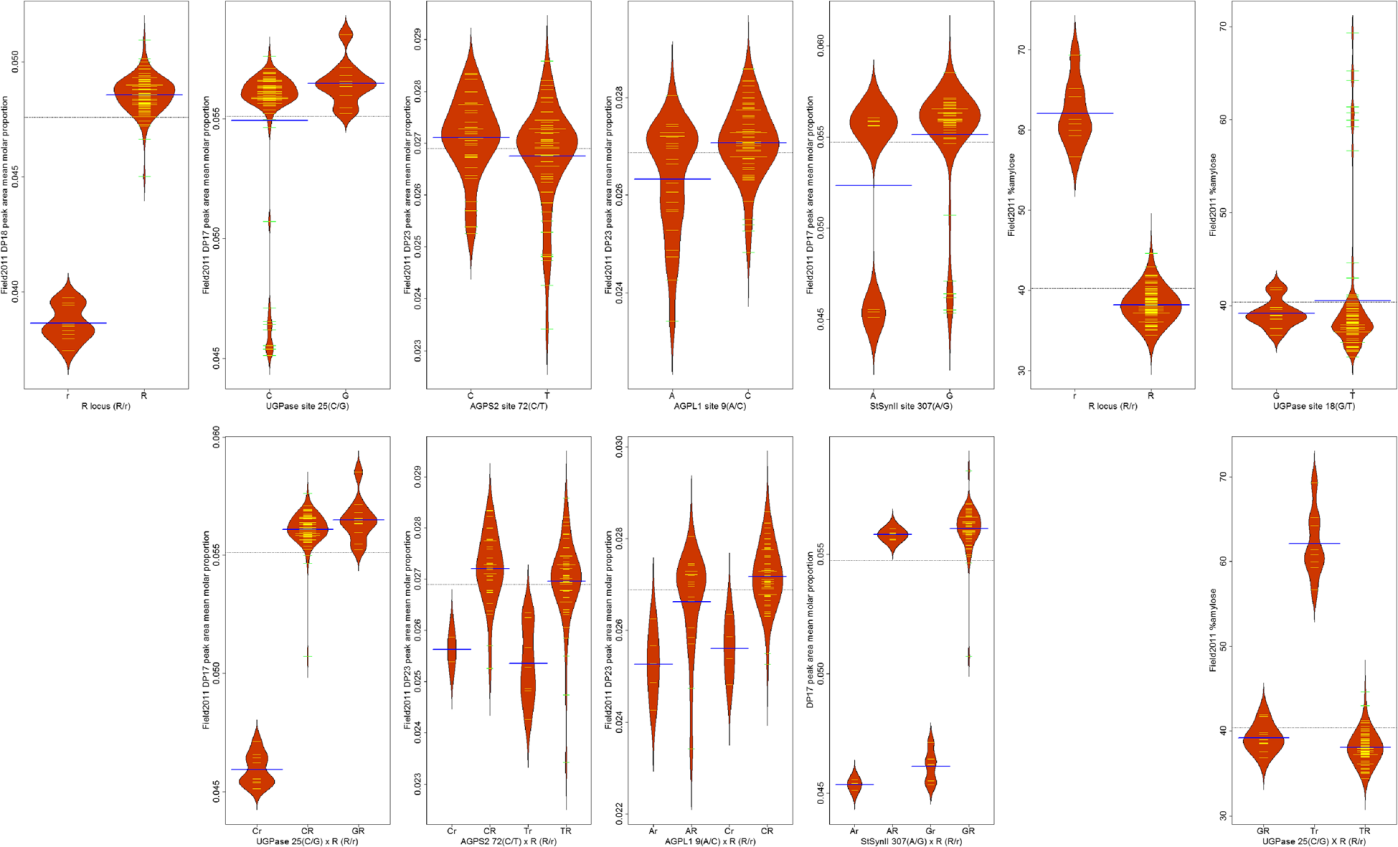
Beanplots showing the distributions of trait values from the Field2011 environment and *n* = 92 PSP lines for alleles of the associated locus-trait combinations**.** The means and distributions of the mean molar peak area proportion values are shown, grouped by allele, or by combinations of alleles from *r* and other loci. Allelic means (*blue lines*) and individual PSP lines’ mean values (*yellow lines*) are shown. The second row contains plots showing pairwise interactions between alleles of *r* locus and the associated allele of the locus directly above each plot

As expected, %amylose was associated with *r* locus in starch from both environments (Table 3, Figs. 5 and 6). In addition, %amylose was associated with polymorphisms in *UGPase* in the Field2011 trial.

**Table 3:** Summary of the most significant associations between candidate carbohydrate and starch metabolism gene sequence polymorphisms versus the percent amylose in extracted starch

Environment: Glasshouse 2010						
Gene	Sequence alignment ^a^	Site on alignment (major/minor allele)	Trait	*p*-value	R^2^ (%)	Q-value
Starch branching enzyme I (*r* locus)	n/a ^b^	n/a	%amylose	2.26 E-33	79.0	4.29 E-31
Environment: Field trial 2011						
Starch branching enzyme I (*r* locus)	n/a	n/a	%amylose	6.85 E-47	86.2	1.90 E-44
UDP glucose pyrophosphorylase	694,186,492	18 (T/G)	%amylose	4.64 E-04	13.1	0.028

^a^GenBank PopSet number

^b^not applicable

Associations were identified using the mixed linear model approach with adjustment for population structure using Q + K matrices, implemented in the Tassel package. Only associations that meet the α < 0.05 criterion for minimising false discovery rate (FDR) are shown

Beanplots are presented summarising the means and distributions of the mean molar peak area proportion values for the allelic variant groups for the significant marker-trait associations for *n* = 92 PSP lines from the GH2010 and Field2011 trials (Figs. 5 and 6). For the associations detected using *n* = 92 PSP lines, the alleles for each associated SNP are presented alone, and also combined with *r* locus, to distinguish the effect of *r* and the other loci, due to the major effect which *r* locus has on CLD profiles. Where four genotypic classes are present, eg. *AGPS2* × *r*, and *AGPL1* × *r*, the plots show pairwise interactions illustrating additive effects between *r* and other loci. In the cases of three other loci (*CWI*, *UGPase*, *SecSuSy*) the pea lines with the wrinkled seeded genotype (*rr*) all fall within a single allelic class of the other locus, therefore only three pairwise genotypic classes occurred.

**Fig. 7:**
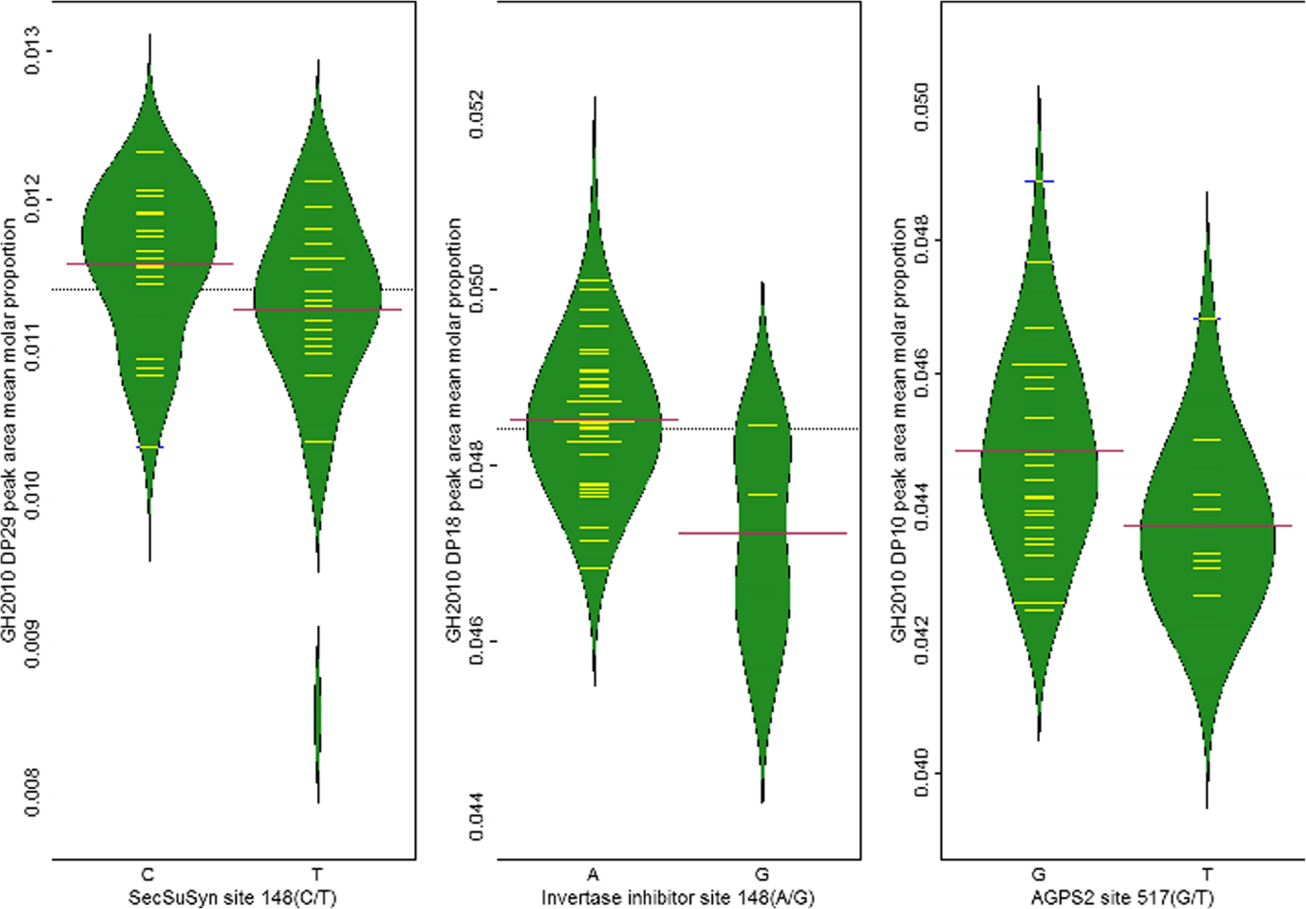
Beanplots showing the distributions of trait values from the Field2011 environment and *n* = 83 round seeded PSP lines for alleles of the associated locus-trait combinations. The means and distributions of the mean molar peak area proportion values are shown, grouped by allele. Allelic means (*red lines*) and individual PSP lines’ mean values (*yellow lines*) are shown

When the subset of 83 round seeded lines were considered, polymorphism in a single candidate gene, *SecSuSy*, was associated at FDR ≤ 0.05 with the DP29 CLD phenotype, in the GH2010 trial (Table 2). If the FDR was relaxed to α < 0.10, then additional associations were identified involving *InvInh* and *AGPS2*, associated with DP18 and DP10, respectively, from the GH2010 trial. Beanplots are presented summarising the means and distributions of the mean peak area proportion values for the allelic variants at the three associated loci for the *n* = 83 lines (Fig. 7). For two other candidate genes, *SPS* (Field2011) and *CWI* (GH2010), polymorphisms associated with CLD were suggested because of low *p*-values (< 0.01) but q-values for these were >0.10. For *SPS*, the polymorphism at site 563 of the Ps_1583 alignment was associated with DP17 (*p* = 7.4 E-04, q-value = 0.171) and for *CWI*, the polymorphism at site 24 of the Ps_0276 alignment was associated with DP39 (*p* = 0.7.9 E-04, q-value = 0.221).

**Fig. 8:**
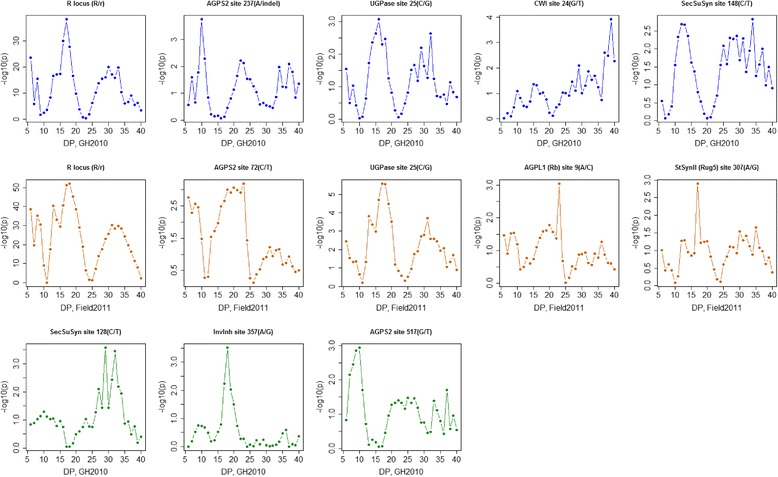
Distribution of *p*-values (as –log_10_(p)) for CLD traits from DP6 to DP40, for candidate gene polymorphisms associated with variation in CLD. The graphs show the *p*-values (as –log10(p)) versus CLD DP values for associated polymorphisms. The top row graphs (*blue*) are plots of DP vs –log10(p) for candidate gene polymorphism associations that meet or exceed the FDR < 0.05 criterion from the GH2010 environment and *n* = 92 PSP lines, and middle row (*red*) are from the Field2011 environment and *n* = 92 PSP lines, containing round and wrinkled seeded PSP lines, while bottom row (*green*) are GH2010 environment locus-trait associations (FDR < 0.10) from the *n* = 83 lines, including only round seeded PSP lines

Associations with low *p*-values were detected between polymorphic sites and the CLD mean molar peak area proportions at a number of DP values. This observation is to be expected since neighbouring DPs can show strong positive correlation and also because mean molar peak area proportions must sum to 1. Hence a change in the abundance of oligosaccharide chains at one DP value or a range of DP values must result in a concomitant change elsewhere in the distribution, which can produce either positive or negative correlations (Additional file 2). To explore further the relationships between variation in candidate gene polymorphisms and the CLD curves, DP versus -log_10_(p) plots were drawn for the *n* = 92 and *n* = 83 populations (Fig. 8). These plots reveal the regions of the CLD curves that were most strongly associated with allelic variation at the significantly associated candidate gene polymorphisms. For the *n* = 92 pea lines and *r* locus, similar profiles were obtained from the GH2010 and Field2011 environments, with *p*-value peaks obtained at DP6, DP17 and around DP30-DP33. Likewise, similar profiles were obtained for the *UGPase* site 25 polymorphism from both environments, with *p*-value peaks at around DP16-DP17 and DP31-DP32. Therefore, the r locus and *UGPase* site 25 polymorphisms affected similar parts of the CLD curves, but this was not due to linkage disequilibrium between these sites (r^2^ = 0.0178). However it does mean that the effects of *r* locus and UGPase on the CLD could be difficult to distinguish. There are also similarities in peak locations for the GH2010 and Field2011 curves obtained for *AGPS2* site 237 and *AGPS2* site 72, although these are less compelling than for *r* locus and *UGPase*.

### Examination of the gene context of polymorphisms

For three of the candidate genes with significant association with CLD traits (*AGPS2*, *UGPase*, *StSynII*) polymorphisms occurred in predicted exon sequences (Table 4). The exonic SNP in *AGPS2* (site 145 in our alignment) resulted in a synonymous codon change. In *UGPase*, the SNP at base 336 of our alignment produced a synonymous codon change and occurred at the 5′ end of an exon, adjacent to an intron /exon boundary predicted by homology with the *M. truncatula UGPase* mRNA (GenBank accession number XM_003616133). In *StSynII* (*rug5*) the SNP at position 307 of our alignment is predicted to produce a non-synonymous change from glycine to serine. This amino acid residue occurs at 249 in the StSynII protein sequence. Analysis of the possible functional effects of this non-synonymous SNP using PROVEAN software predicted it to be “neutral”, with a score of −0.244 where a score < −2.5 predicts that a variant is likely to be “deleterious”. A significantly associated SNP in *AGPL1* (at position 9) occurred in the 5′ UTR of the *AGPL1* mRNA. The remaining polymorphisms in the candidate gene sequences that were significantly associated with starch traits occurred in intron sequences.

**Table 4:** Exonic or 5′ UTR polymorphisms significantly associated with chain length distribution (CLD) trait variation in pea lines and their predicted effects on translation product sequence

Candidate gene	Alignment PopSet number	Site on alignment	Variant	Effects (site on reference sequence translation)
AGPS2	694,187,965	145	T/C	In exon; CTT / CTC, leucine -> leucine, synonymous
UGPase	694,186,492	336	T/C	Variant in the first base of the exon, adjacent to a predicted intron acceptor site; codon change AGT / AGC, serine -> serine, synonymous
Starch synthase II (*rug5*)	1,206,484,033	307	G/A	In exon; GGT / AGT, glycine -> serine (249), non-synonymous
AGPL1 (*rb*)	694,185,424	9	A/C	In 5′ UTR

## Discussion

### Phenotypic analysis

This study has focused on variation in two pea starch characteristics, %amylose and the CLD of debranched extracted total pea starch, as determined by quantitative FACE. Although total starch was the starting material for the FACE, analysis of CLD in debranched starch in the DP range used in this study (DP6 – DP40) is generally considered to examine the amylopectin fraction, since short (mean DP ~ 15) and medium length (mean DP ~45) chains derive from amylopectin [51, 52]. In association mapping studies a relatively large number of lines must be both phenotyped and genotyped, with phenotyping based on trialling in multiple environments and using appropriate trial design and replication. Analysis of starch structural and functional properties requires a number of labor-intensive preparative and analytical steps. Starch extraction in particular is time consuming. The need to extract starch from seeds from approximately 500 samples made it necessary to start with relatively small amounts of seeds (5 g) from each trial pot (GH2010) or plot (Field2011) and consequently starch yields were small. Hence, this research focused on understanding CLD using FACE, a method that requires only a small amount of starch and is suitable for moderate throughput.

A limited number of studies have examined CLD variation in debranched pea starch. For example, Ratnayake et al. [53] explored the differences in CLD in four field pea (round seed) lines and observed differences in the relative abundance of the DP6 peak, the DP for the largest peak of the distributions, and the shapes of the distributions in the DP16 to DP26 regions. Variation in amylose content has also been explored in small numbers of round peas [13, 14].

### Population structure estimation

False associations are a potential difficulty with association mapping studies using germplasm panels because of unknown relatedness or population structure [31, 54]. As a result, apparent marker-trait association may occur when trait values and marker allele frequencies are correlated based on subpopulation or kinship, rather than being due to linkage between quantitative trait loci (QTL) and markers, leading to spurious associations. Mixed model methods are used to relate the relatedness matrix (Q, P, and/or K) to a phenotype, yielding a relatedness-based weighted average predicted phenotype.

Determination of the Q, P or K matrices relies on polymorphism information from random molecular markers that are distributed throughout the genome. In our study, we used relatively few markers (*n* = 55) and a mixture of marker types (SSRs, SCARs, and RAPD polymorphisms) that revealed 140 polymorphisms. Since our panel consisted of single-seed derived inbred lines, all markers, including RAPDs, provided homozygous genotypes. Using these markers we estimated the relatedness of pea lines to provide Q (Fig. 4), P and K matrices. Population structure estimation using STRUCTURE and PCA gave a clear answer of three subpopulations.

Prior to this study, the estimation of population structure in pea has been undertaken in collections of diverse germplasm, including studies of the John Innes *Pisum* germplasm [55], the USDA pea core collection [56], and the European germplasm collections [57]. Each of these studies estimated three populations within their respective germplasm collections, and further subdivision of populations was also indicated for the John Innes collection and European germplasm [55, 57]. The study of the USDA pea core (*n* = 285 lines) used a relatively small number of markers from a combination of SSRs (15 primer pairs), RAPDs (36 loci) and one SCAR [56], while the John Innes (*n* = 3020 lines) and European germplasm (*n* = 4538) studies relied most heavily on retrotransposon-based polymorphism markers (RBIP and SSAP), also in relatively small numbers, 45 and 27 respectively. Jing et al. [57] expressed confidence in their population structure estimation because similar results were obtained from both RBIP and SSAP marker types.

### Allelic variation and association with starch physicochemical properties

In this study, we focused on association mapping of variation in debranched starch CLD and %amylose with polymorphisms in 25 candidate genes representing 16 carbohydrate and starch metabolic enzymatic reaction classes (Table 1, Fig. 3). Using the total population of *n* = 92 PSP lines, associations that met the FDR α ≤ 0.05 criterion were identified for polymorphisms in seven pathway genes, representing six of the enzymatic reaction classes in Fig. 3. For the round seeded PSP lines (*n* = 83), associations that met the FDR α ≤ 0.10 criterion identified polymorphisms in three pathway genes. Taken together, eight pathway genes were associated with CLD variation and these associated candidate genes were involved in substrate availability [*CWI*, *SecSuSy*, *AGPS2*, *AGPL1* (*rb*), *UGPase* and *InvInh*], chain elongation [*StSynII* (*rug5*)] and branching [*SBEI* (*r*)].

The power of our study to identify sequence variants associated with starch CLD variation was limited. The limiting factors included: 1) the relatively small sample size (*n* = 92) we employed, 2) the limited number (25) of candidate genes in the starch biosynthetic pathway that were characterised, and 3) that the candidate genes were partially sequenced, with a bias for intron-containing regions, therefore causal variants may have occurred outside the sequenced regions and may have been in only partial LD with variants within the sequenced regions. The genome-wide association study (GWAS) offers an alternate approach to the candidate gene-based approach for identifying genes associated with complex traits such as the composition of pea seeds, although GWAS is poor for detecting effects of minor alleles [58]. Substantially larger sample size is needed to increase the statistical power to detect effects, and methods for accurate phenotyping of pea seed compositional traits need to be appropriate for the increased throughput that is required.

CLD affects gelatinization and pasting properties of starch [8, 9]. As a generalization, an increased proportion of short chains (DP6 – DP12) results in reduced gelatinization temperature and enthalpy, and also reduces pasting temperatures and viscosities, while increased proportions of intermediate (DP13 – DP24) and long (>DP24) chains increase gelatinization temperature and enthalpy as well as pasting temperatures. The largest effect on amylopectin CLD in our study was obtained from the round (*RR*) versus wrinkled (*rr*) lines. The mean proportion of intermediate length chains was increased in round seeded lines (Fig. 8) with an associated decrease in the mean proportion of short and long chains (data not shown). Therefore, it is difficult to predict the overall effect that the *r* locus allelic variation would have on the thermal and pasting properties of pea amylopectin. Allelic variation in *UGPase* (at site 25, both trials) also affected the mean proportion of intermediate length chains, which was higher on average for the lines with the G allele at that site.

Mutational studies in pea have demonstrated the role of six pea seed starch biosynthetic genes in pea seed starch accumulation, structure and function [5, 59, 60]. In double mutant nearly isogenic lines, Lloyd et al. [60] found a modest effect on CLD of *r* locus (*SBEI*), and a smaller effect of r*b* locus (*AGPL1*) mutations. Mutations in *rug5* (*StSynII*) were also found to affect CLD in both developing and mature pea embryos [5, 59]. In the present study, naturally occurring allelic variation in *r*, *rb* (*AGPL1*) and *rug5* (*StSynII*) was associated with CLD variation (Table 2, Figs. 5 and 6). However, in our germplasm panel, we failed to detect association between CLD and polymorphisms in *PGMP* (*rug3*), *GBSSI* (*lam*) and *SuSy* (*rug4*), the other three genes identified, based on EMS mutations, in the above studies as being involved in starch metabolism.

Variation in %amylose was associated with allelic variation in *r* locus, the major gene responsible for the difference between round and wrinkled (high amylose) pea lines, in both trials, as expected. In starch extracted from plots from the Field2011 trial, %amylose was also associated with SNPs in *UPGase*. For the GH2010 trial, %amylose was only associated with *r* locus. This likely reflects the greater variation in %amylose observed among round seeded lines in the Field2011 trial than the GH2010 trial, in which variation between round seeded lines was not significant. The effects of mutations in pea starch biosynthetic genes on %amylose have previously been determined, with *rug3* (*PGMP*) and *lam* (*GBSSI*) reducing the % amylose content of starch compared with the wild type round seeded line, while *rug5* (*StSynII*) and *r* (*SBEI*) mutants increased the percent amylose [16]. In a recent study of 50 round seeded pea lines, Jha et al. [61] have found association (0.01 > *p* > 0.001) of amylose content with SNPs in amplified fragments of the *AGPL1* (*rb* locus), *GBSSI* (*lam* locus) and *SBEII* genes. However, we did not detect association in %amylose involving SNPs in these genes. This difference that may be due to the germplasm used in the different studies or to the relatively low power of both studies due to the numbers of lines used.

As CLD and amylose content affect the physicochemical properties of starches, there are parallels, as well as contradictions, between our results and those of association mapping studies which looked at physicochemical properties of starches from other species. In pea, *SBEI* (*r* locus) had the greatest effect on both amylose content and CLD, whereas in rice, polymorphisms in *GBSS* (*Wx*) had the greatest effect on amylose content, and *SSII* on gelatinization temperature [23], with *SBE3* having only a minor effect on gelatinization temperature. In maize, polymorphisms in a sucrose synthase gene (*sh1*), *AGPL1* (*sh2*) and *SBEIIb* (*ae1*) were associated with amylose content and pasting properties [24], while in pea, polymorphisms in genes from these families were associated with CLD and/or amylose content. A study in sorghum detected polymorphisms in *SSII* and *SBE* associated with physicochemical properties, similar to the results for CLD in pea, but differed from pea in that association with *GBSS* was also found [26]. Where the results from different species are contradictory, this reflects the fact that the associations which can be detected are limited by the variation which occurs in any germplasm collection, and by the power of the experiment.

Analysis of the gene context of the polymorphisms associated (FDR α ≤ 0.05) with CLD showed that most of these occurred in intronic sequences. For three of the genes, associated polymorphisms occurred in exonic regions (*AGPS2*, *UGPase*, *StSynII*), although only the mutation in *StSynII* resulted in a non-synonymous codon change (Table 4). Analysis of the predicted effect of the codon change in *StSynII* (residue 249, glycine or serine) on protein function using PROVEAN software [39] indicated that the effects were most likely to be neutral. For the *UGPase* gene, one of the significantly associated polymorphic SNPs occurred immediately upstream of a predicted intron acceptor cut site, hence may affect transcript splicing in lines with one or the other of the variants, a possibility that is able to be tested experimentally. Therefore, with the possible exception of the intron acceptor site variation in the *UGPase* gene, it is unclear whether any of the significantly associated polymorphisms identified are directly responsible for changes that would affect the CLD phenotypic variation, either through changes in coding or non-coding regions. Since only portions of the candidate genes were characterized for sequence polymorphism, it is quite possible that the significantly associated polymorphisms that were detected are in LD with the causal mutation(s) to be found in the gene regions that were not sequence characterized. It is also possible that the causal variants underlying the CLD traits do not occur in the candidate genes but in linked sequences. Our analysis of the decay of LD in this population has been limited to examining the LD within individual fragment alignments (where the maximum pairwise LD (r^2^) within fragments was 1.0 or nearly), which is of limited value because of the small size of these fragments, and to examining pairwise LD in pairs of fragments amplified from seven of the pea genes characterized (Additional file 3). While the actual physical distances between the polymorphisms in the paired fragments are unknown, their relative positions on the mRNA sequences are known. A range of LD conservation was observed; seen for example by contrasting LD decay in *StSynII* (where the maximum pairwise r^2^between genomic fragments from 587 to 999 and 1809–2229 on the mRNA was 0.173) with *UGPase* (where the maximum pairwise r^2^ between genomic fragments from 59 to 201 and 1529–1601 on the mRNA was 0.833).

## Conclusions

We have shown that allelic variation in pea starch pathway candidate genes can have a measurable effect on amylopectin CLD. We identified polymorphisms in eight genes from the pea seed carbohydrate and starch metabolic pathway as having significant association (FDR ≤ 0.05 or FDR ≤ 0.10) with variation in debranched starch CLD, and two genes (*r* locus and *UGPase*) as having significant association with variation in %amylose. The findings were based on analysis of seed starch extracted from 92 diverse pea lines grown in two replicated trials, and on polymorphisms detected by sequencing fragments of 25 candidate genes and by scoring *r* locus. The *r* locus, which encodes SBEI, had the major effect (R^2^ of 83.4–88.6%), but other genes involved in substrate availability (*CWI*, *InvInh*, *SecSuSy*, *AGPL1, AGPS2*, and *UGPase*) and chain elongation (*StSynII*) were also associated with CLD variation, with effects ranging from 11.8 to 22.2% of the variation in mean peak area proportion at the most strongly associated peak, as determined by quantitative FACE. Examination of the sequence context of the significantly associated polymorphisms showed that most occurred in intronic regions, but a polymorphism in *UGPase* occurred immediately upstream of a predicted intron 3′ acceptor cut site, and a polymorphism in *StSynII* produced a non-synonymous mutation that was most likely to have a neutral effect on protein function. Hence, the candidate gene polymorphisms causing CLD variation in pea seed starch are in most if not all cases likely to be in full or partial LD with the associated polymorphisms that we have detected, and these causal variants may either occur within the candidate gene or in nearby sequences. Nevertheless, this study identifies sequence polymorphisms in carbohydrate and starch pathway genes, and publicly available pea lines containing the allelic variants, that can be used for further studies of genetic determination of pea seed starch structure and function, including plant breeding.

## Additional files

Additional file 1: Pea single plant (PSP) accessions used for association mapping with Names and Collection Country from the USDA-ARS GRIN database (https://npgsweb.ars-grin.gov/gringlobal/search.aspx), *r* locus phenotype (1 = round, 0 = wrinkled seeded), % amylose and CLD mean molar peak area proportions for the Field2011 and GH2010 environments. (XLSX 136 kb)

Additional file 2: Heat maps showing correlations between chain length distribution (CLD) mean peak area proportions for debranched starch from peas grown in GH2010 versus Field2011 trials. The colour scale ranges from blue (more strongly positive correlations) to yellow (more strongly negative correlations). The top panel shows correlations for all *n* = 92 lines (round and wrinkled seed) while the bottom panel shown correlations for *n* = 83 round seed lines only. (PNG 251 kb)

Additional file 3: Heat map showing the extent of linkage disequilibrium (r^2^) within and among polymorphisms in 32 genomic fragments representing 25 candidate genes and *r* locus. Abbreviated candidate gene names are shown along the diagonal of the Figure. (PNG 56 kb)

Additional file 4: Principle component analysis of population structure for *n* = 92 PI PSP lines using 55 background markers. (A) Principle components biplot for the first two PCs. Round seeded lines are indicated with clear circles and wrinkle seeded lines with red circles. (B) Scree plot of eigenvalues showing the variation in each component. (PNG 77 kb)

Additional file 5: Q-Q plots of the observed versus expected -log_10_(P) for the DP-environment combinations which gave associations with the lowest *p*-values. Results for the MLM + Q + K model (blue) and MLM + P + K model (red) are shown. The DP-environment combinations are indicated on the y-axes. (PNG 34 kb)

## Abbreviations

%amylose: Percent amylose
ae: Amylose extender
AGPL1: ADP-glucose pyrophosphorylase L1 subunit
AGPS2: ADP-glucose pyrophosphorylase S2 subunit
APTS: 8-aminonaphthalene-1, 3, 6-pyrenetrisulfonic acid
BAM: Beta-amylase
CLD: Chain length distribution
CWI: Cell wall invertase
DMSO: Dimethyl sulfoxide
DP: Degrees of polymerization
FACE: Fluorophore-assisted carbohydrate electrophoresis
Field2011: Field trial held over the New Zealand summer of 2011–2012
GBSS: Granule bound starch synthase
GH2010: Glasshouse trial held in 2010–2011
Hex: Hexokinase
InvInh: Invertase inhibitor
ISA: Isoamylase
LD: Linkage disequilibrium
MLM: Mixed linear model
PCA: Principal components analysis
PCR: Polymerase chain reaction
PGMP: Plastidial phosphoglucomutase
PSP: Pea single plant
PUL: Pullulanase
PWD: Phosphoglucan water dikinase
QTL: Quantitative trait locus/loci
RAPD: Random amplified polymorphic DNA
RFU: Relative fluorescent units
SBE: Starch branching enzyme
SCAR: Sequence characterized amplified region
SecSuSy: Second sucrose synthase
SPS: Sucrose phosphate synthase
SSR: Simple sequence repeat
StSyn: Starch synthase
SuSy: Sucrose synthase
UGPase: UDP-glucose pyrophosphorylase
