# Supplementary figures and images for: Association mapping of starch chain length distribution and amylose content in pea (Pisum sativum L.) using carbohydrate metabolism candidate genes
Source: BMC Plant Biol. 2017 Aug 1;17:132. doi: 10.1186/s12870-017-1080-9 (PMC5540500; doi:10.1186/s12870-017-1080-9)

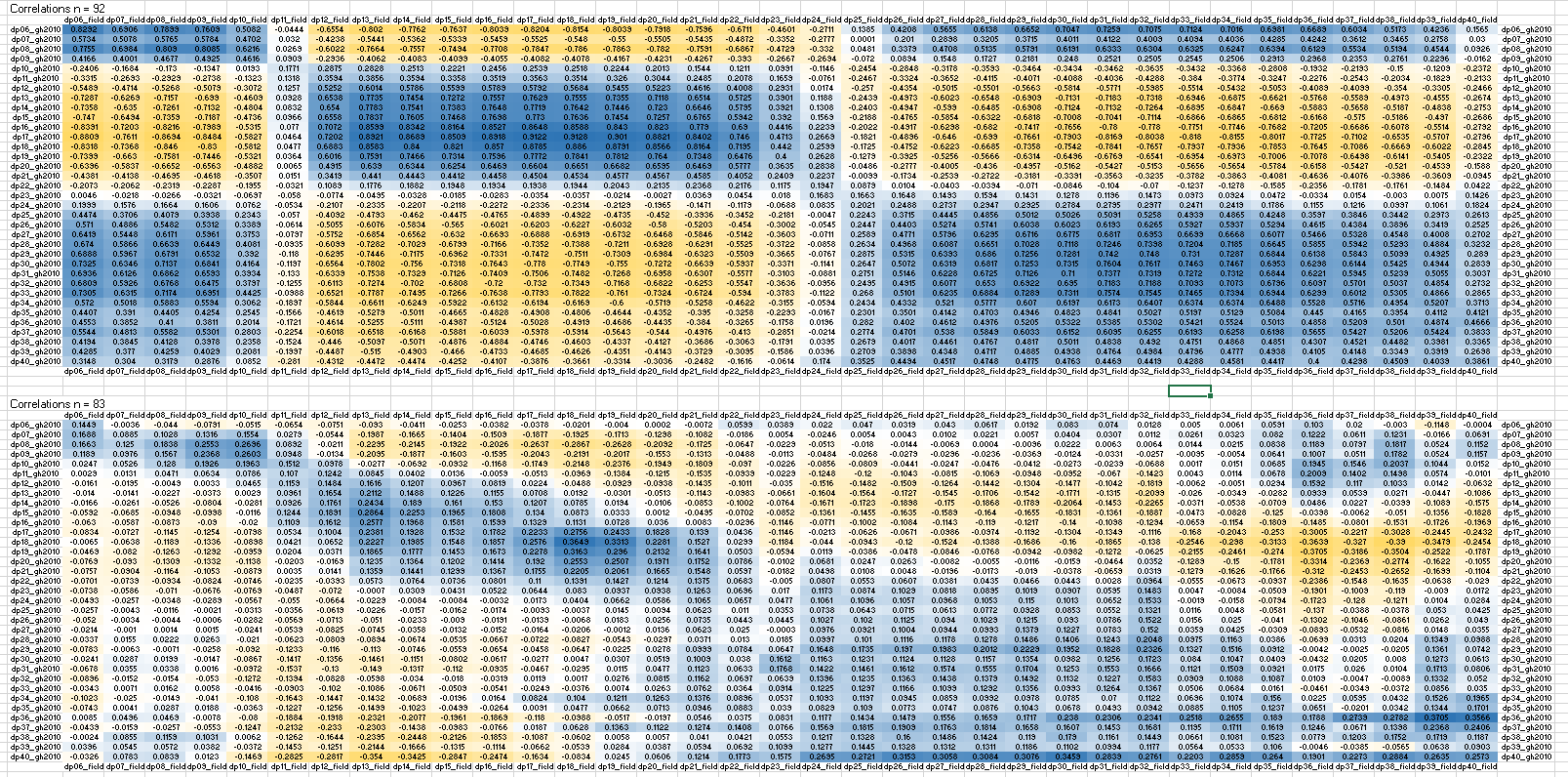

Supplement: Supplementary file 2 — Heat maps showing correlations between chain length distribution (CLD) mean peak area proportions for debranched starch from peas grown in GH2010 versus Field2011 trials. The colour scale ranges from blue (more strongly positive correlations) to yellow (more strongly negative correlations). The top panel shows correlations for all n = 92 lines (round and wrinkled seed) while the bottom panel shown correlations for n = 83 round seed lines only. (PNG 251 kb) [file 12870_2017_1080_MOESM2_ESM.png]

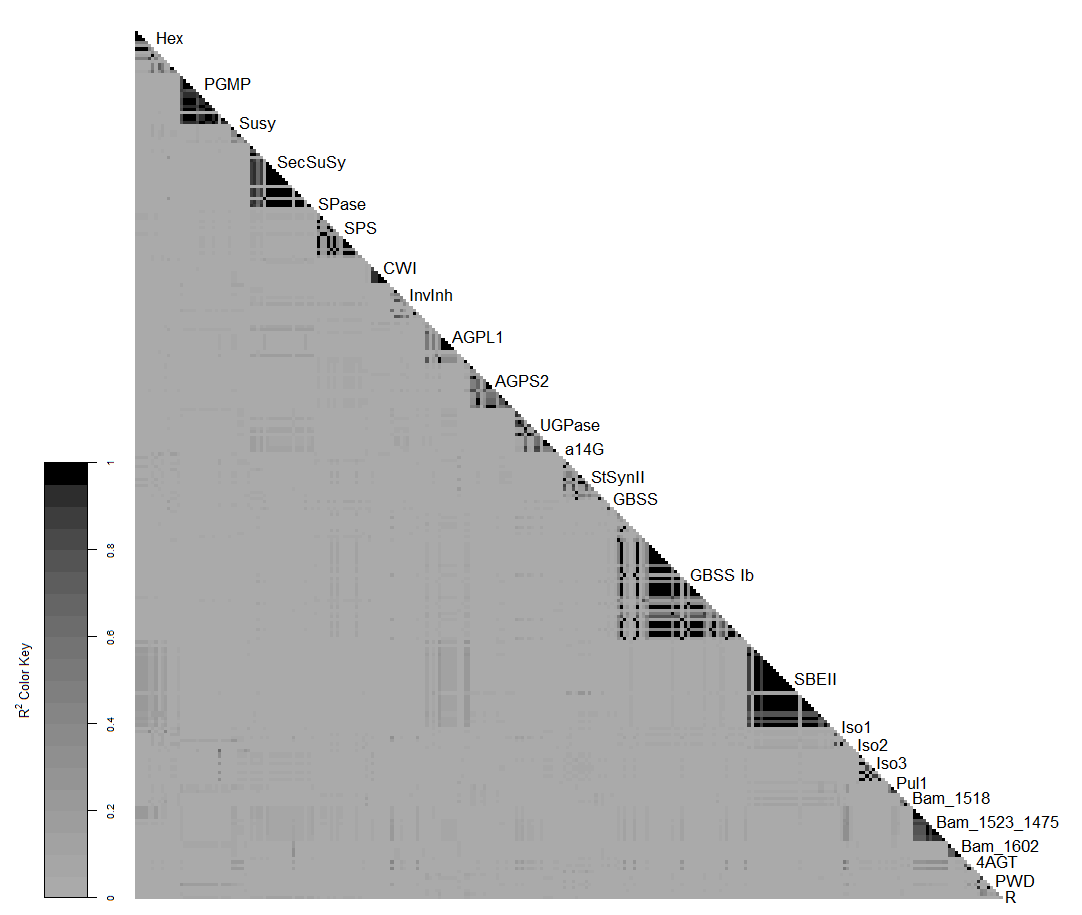

Supplement: Supplementary file 3 — Heat map showing the extent of linkage disequilibrium (r2) within and among polymorphisms in 32 genomic fragments representing 25 candidate genes and r locus. Abbreviated candidate gene names are shown along the diagonal of the Figure. (PNG 56 kb) [file 12870_2017_1080_MOESM3_ESM.png]

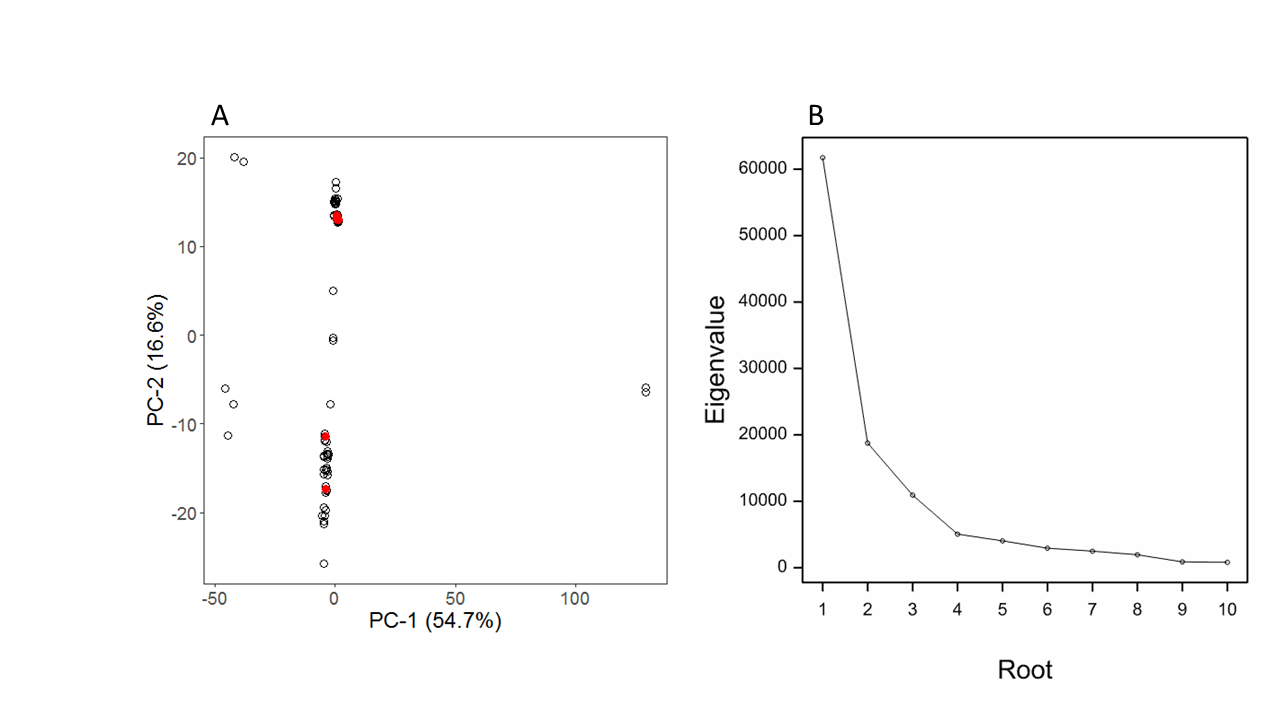

Supplement: Supplementary file 4 — Principle component analysis of population structure for n = 92 PI PSP lines using 55 background markers. (A) Principle components biplot for the first two PCs. Round seeded lines are indicated with clear circles and wrinkle seeded lines with red circles. (B) Scree plot of eigenvalues showing the variation in each component. (PNG 77 kb) [file 12870_2017_1080_MOESM4_ESM.png]

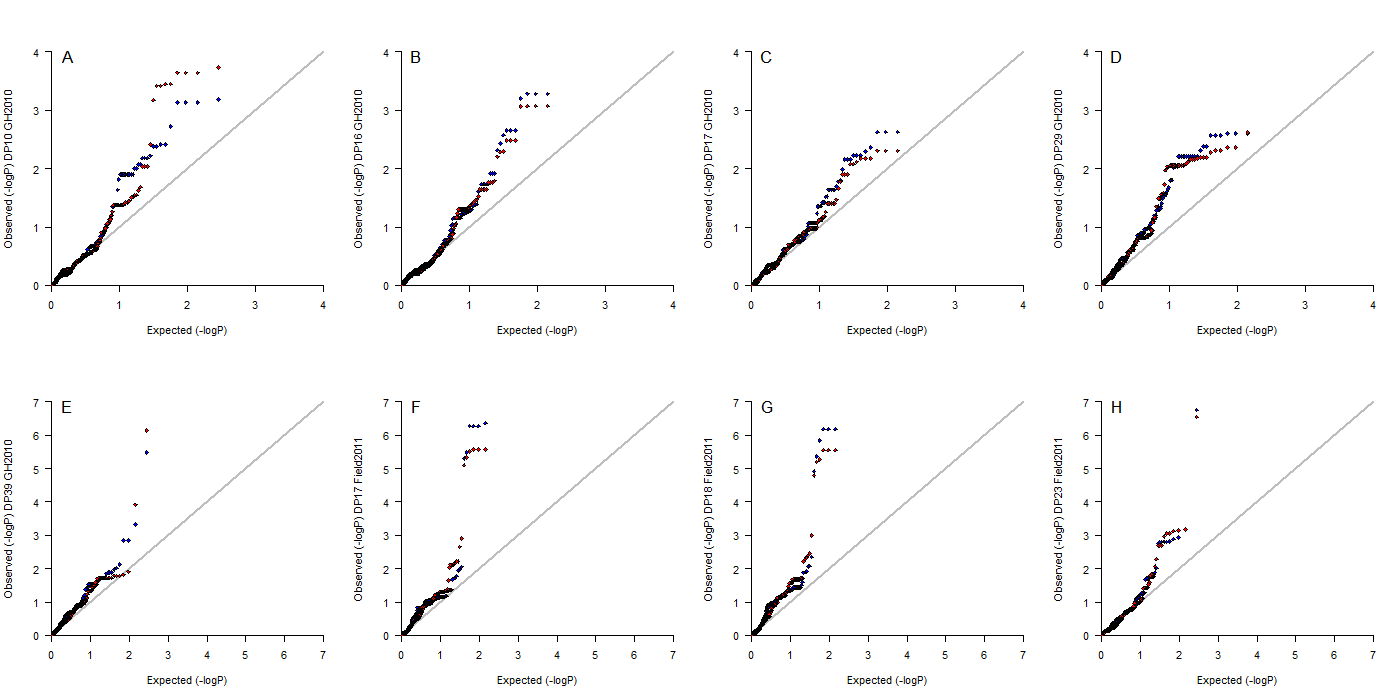

Supplement: Supplementary file 5 — Q-Q plots of the observed versus expected -log10(P) for the DP-environment combinations which gave associations with the lowest p-values. Results for the MLM + Q + K model (blue) and MLM + P + K model (red) are shown. The DP-environment combinations are indicated on the y-axes. (PNG 34 kb) [file 12870_2017_1080_MOESM5_ESM.png]
